# Enhanced Two-Step Extraction from Biomass of Two *Cymbopogon* Species Cultivated in Santander, Colombia

**DOI:** 10.3390/molecules28176315

**Published:** 2023-08-29

**Authors:** Angie K. Romero, Daysy J. Portillo, Sheila B. Beltrán, Lady J. Sierra, Camilo A. Álvarez, Karen J. Ramírez, Jairo R. Martínez, Elena E. Stashenko

**Affiliations:** 1 Research Center for Chromatography and Mass Spectrometry (CROM-MASS), Universidad Industrial de Santander, Bucaramanga 680002, Colombia; cenivam.sgr03@uis.edu.co (A.K.R.); cenivam.sgr01@uis.edu.co (D.J.P.); sheila2218097@correo.uis.edu.co (S.B.B.); lady.sierra2@correo.uis.edu.co (L.J.S.); jmartine@uis.edu.co (J.R.M.); 2 Research Center for Biomolecules (CIBIMOL), Universidad Industrial de Santander, Bucaramanga 680002, Colombia; camilo.alvarez@uis.edu.co (C.A.Á.); karen2188739@uis.edu.co (K.J.R.)

**Keywords:** essential oil, *Cymbopogon*, LC/MS, ORAC, ABTS, circular economy

## Abstract

The insertion of circular economy principles into the essential oil (EO) production chain aims to reduce waste generation and make integral use of harvested plant material. Higher profits from integral use with reduced waste generation contribute to the eventual use of the EO value chain as an alternative to illicit crops in Colombia (mostly coca). In this study, Java-type citronella (*Cymbopogon winterianus*) and palmarosa (*C*. *martinii*) plant materials were used in two consecutive processes to obtain EOs and extracts. The residual biomass after EO distillation was subjected to ultrasound-assisted hydroethanolic extraction to afford extracts that contained bioactive compounds. Citronella and palmarosa were distilled with typical EO yields (1.0 ± 0.1% for citronella; 0.41 ± 0.06% for palmarosa; *n* = 5) either through hydrodistillation assisted by microwave radiation or through steam distillation, and their composition (determined via GC/FID/MS analysis) and physicochemical parameters fell within their ISO standard specifications. The concentration of citronellal, the major compound of citronella oil, was 500 ± 152 mg/g. Geraniol, the main component of palmarosa oil, was found at 900 ± 55 mg/g. The citronella and palmarosa hydroalcoholic extracts (4–11% yield) were analyzed with UHPLC-ESI-Orbitrap-MS, which permitted the identification of 30 compounds, mainly C-glycosylated flavones and hydroxycinnamic acids. Both extracts had similar antioxidant activity values, evaluated using the ABTS^+●^ and ORAC assays (110 ± 44 µmol Trolox^®^/g extract and 1300 ± 141 µmol Trolox^®^/g extract, respectively).

## 1. Introduction

The *Cymbopogon* genus of the Poaceae (Gramineae) family is made up of 140 species, mainly represented by grasses and aromatic plants that are industrially distilled to obtain essential oils, rich in oxygenated compounds such as geraniol, citronellol, citronellal, and citral, with diverse biological activity [1,2]. *Cymbopogon martinii* (palmarosa) and C. *winterianus* (Java-type citronella) are perennial plants native to South Asia and are grown in tropical and subtropical regions, mainly in India, Sri Lanka, Pakistan, Brazil, and Paraguay [3].

Citronella and palmarosa EOs are widely used as raw materials in the production of perfumes, soaps, cosmetics, insecticides, fragrances, detergents, and pharmaceuticals [4,5,6,7,8,9,10,11]. The EOs are obtained through steam distillation (S.D.) or hydrodistillation (HD), and it is expected that, by 2025, the cultivated area of citronella worldwide will reach 60 thousand hectares [12]. Variations in agronomic [13,14], environmental, and distillation conditions [15] can generate changes in the quality parameters of EOs, which are regulated by the international standard ISO 3848 of 2016 [16] for Java-type citronella and by ISO 4727 of 2021 [17] for palmarosa.

The EO agroindustry generates a very large amount of residual biomass. Approximately 98% of plant material becomes a byproduct that is used as compost [18] or biofuel [19] or is landfill discarded. Residual plant material still contains compounds of biological and industrial interest, including flavonoids, which do not decompose at the temperatures of the steam and hydrodistillation processes [20,21,22]. The use of residual biomass to obtain extracts containing bioactive molecules and the subsequent utilization of the extraction residue for composting or biochar generation provides additional income and reduces disposal costs in the production chain. This sustainable approach enhances the competitiveness of the EO agroindustry and the possibility of becoming an alternative to illicit crops in Colombia (mostly coca).

The role played by flavonoids in plants is related to their defense mechanism against pathogens and insects or the attraction of pollinators [23]. Flavonoids are widely studied for their biological properties [23,24]. Several authors [25,26,27,28,29] have reported on flavonoids present in extracts from different *Cymbopogon* sp. plants before distillation; however, studies on the pharmacological and phytochemical properties of these extracts are still very scarce. 

In this work, it was determined that EOs distilled from Java-type citronella and palmarosa plants grown in five municipalities of Santander, Colombia, meet the technical specifications, chemical compositions, and physicochemical properties established by ISO standards [16,17] for their trade. It was found that the residual biomass, after the plant material distillation process, was a source of phenolic compounds, whose antioxidant properties were then studied. In Santander, the distillation of EOs and obtaining extracts from aromatic plants of *Cymbopogon* spp. could be a new way in the regional economic sector to produce natural ingredients important in many products of the cosmetic and pharmaceutical industries and, above all, as insect repellents for those vectors responsible for various diseases (malaria, dengue, yellow fever, chikungunya, and zika, among others) widespread in hot and humid climates, typical of the Colombian countryside.

## 2. Results

### 2.1. Essential Oil Yields

Java-type citronella and palmarosa were harvested from plantations located in Bucaramanga (*n* = 5), Barbosa, Chipatá, Puente Nacional (*n* = 3), and Vélez (*n* = 2–3) from March to December 2022. The yields of the EOs obtained via the microwave-assisted hydrodistillation (MWHD) and steam distillation methods from plants cultivated in five municipalities of Santander (Barbosa, Bucaramanga, Chipatá, Puente Nacional, and Vélez) are shown in Table 1. The EO average yields of citronella (1.0 ± 0.2%) were higher than those obtained for palmarosa (0.4 ± 0.1%) using either MWHD or S.D., independent of the municipality that provided the plant material. The plant material employed for MWHD and S.D. was fresh. The phenological stage of Java-type citronella was vegetative, while that of palmarosa was post-flowering. The partial superposition of the uncertainty corridors (standard deviations) of the average yields (Table 1) indicated that there were no significant effects with respect to the essential oil isolation technique employed (MWHD or S.D.) or the plantation location.

### 2.2. Chemical Composition of Essential Oils Distilled via MWHD from Plants Harvested from Different Santander Municipalities 

**Figure 1:**
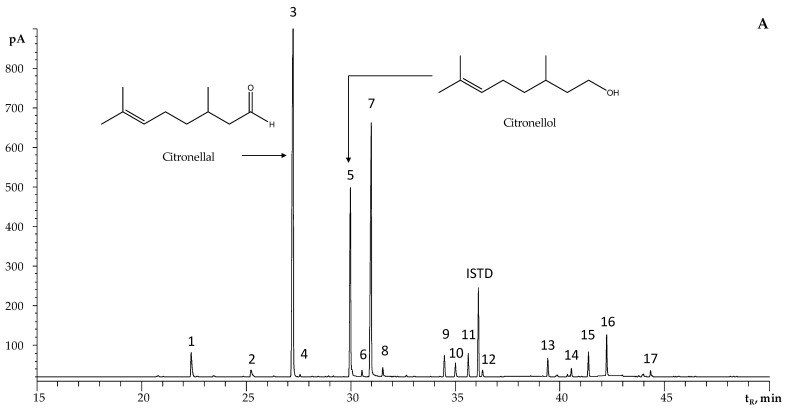

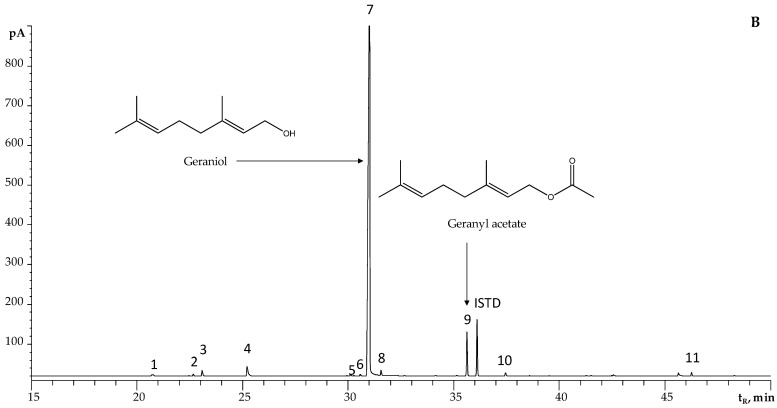
Chromatographic profiles obtained using the GC/FID of *Cymbopogon* sp. EOs. DB-5MS column (60 m). Split, 1:30. ISTD: *n*-tetradecane (0.5 g/L). (**A**) Java-type citronella (*C. winterianus*); (**B**) palmarosa (*C. martinii*). See peak identification in Table 2 and Table 3.

The chromatographic profiles of the EOs of Java-type citronella and palmarosa are presented in Figure 1, and the chemical compositions of the EOs distilled from plants harvested from the different municipalities of Santander are described in Table 2 and Table 3. The GC/FID/MS analysis of the EOs of Java-type citronella under study allowed for the identification of 17 compounds in relative amounts > 0.1%, mainly oxygenated monoterpenes (85%) and, to a lesser extent, oxygenated sesquiterpenes (9%) and sesquiterpene hydrocarbons (3%); limonene (1.5%) was the only monoterpene hydrocarbon detected. The EOs from palmarosa contained 11 terpenes (>0.1%); among them, geraniol (83–87%), geranyl acetate (4–7%), and linalool (1.7–3.9%) were the major components.

The quantification of the compounds in the EOs of Java-type citronella and palmarosa was carried out using the external standard calibration method. Tables S3 and S5 (Supplementary Materials) show the results of the quantification of the main compounds identified in the EOs of citronella and palmarosa. The results are expressed as mg substance/kg EO ± standard deviation. In the citronella EO, the concentration of citronellal, the major compound, was 320–650 mg/g EO. In the EO of palmarosa, the major compound was geraniol (830–960 mg/g EO).

### 2.3. Physicochemical Essential Oil Parameters 

The plants under study were harvested from plantations located in the five municipalities of Santander from March to December 2022 (*n* = 16). The plant material was homogenized at the collection center in Barbosa, Santander, and subjected to steam distillation. The EO yields of Java-type citronella and palmarosa obtained through S.D. were 0.8 ± 0.1% and 0.37 ± 0.06%, respectively. The physicochemical properties of the EOs of Java-type citronella and palmarosa are presented in Table S7, together with the corresponding values in the ISO 3848:2016 and ISO 4727:2021 standards for citronella and palmarosa EOs, respectively. Both EOs met the expected intervals of the ISO standards. Their chemical compositions appear in Table S8.

### 2.4. Chemical Composition of Java-Type Citronella and Palmarosa Hydroalcoholic Extracts

No significant differences were found for the hydroalcoholic extraction yields from the undistilled plant material compared with those from the residual biomass after the distillation of either Java-type citronella or palmarosa (Tables S9 and S10).

**Figure 2:**
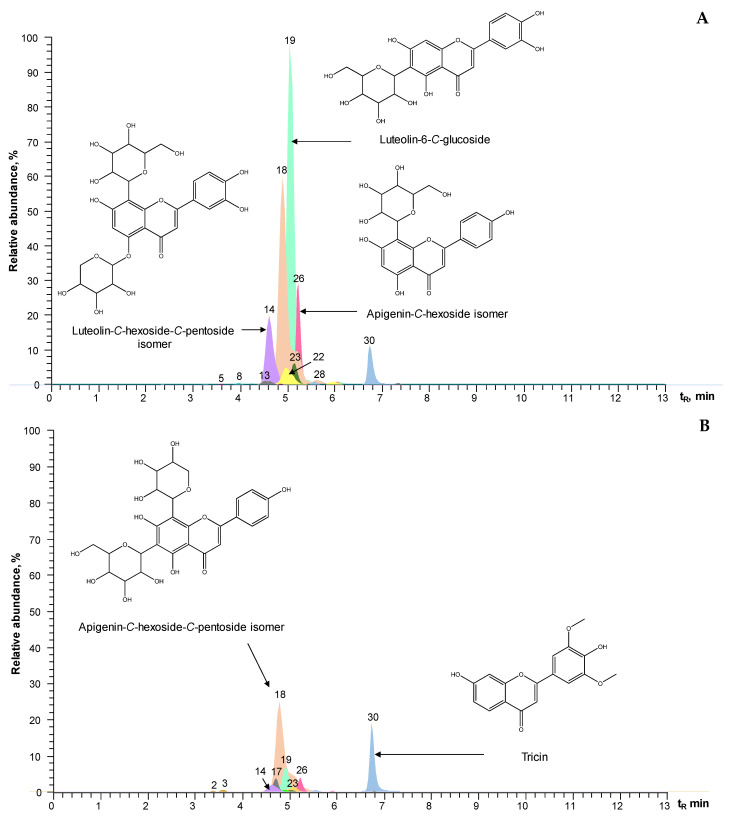
Extracted ion currents (EICs), obtained using UHPLC-ESI^+/−^-Orbitrap-MS, operated in SIM mode to filter the protonated [M + H]^+^ or deprotonated [M − H]^−^ molecules of substances present in *C. martinii* solvent extracts isolated from (**A**) plant material prior to distillation and (**B**) postdistillation waste. Scale 6.8 × 10^7^. See peak identification in Table S6.

The extracted ion currents (EICs) of the protonated or deprotonated molecules, obtained in the dual acquisition of positive and negative ions, prefiltered with a quadrupole, are shown in Figure 2. The exact masses of the detected substances (calculated and experimental), their elemental compositions, the mass measurement error for each compound (∆ ppm < 3), and the product ions used for the identification of the molecules are reported in Table S11. Mass spectra were obtained by applying different energies in the higher-energy collision dissociation cell (HCD, 10–70 eV). Compound identification was based on the exact masses of protonated [M + H]^+^ or deprotonated [M − H]^−^ molecules based on the study of their fragmentation pattern (product ions) and isotopic ratio and on the use of reference substances and spectral databases, i.e., the Human Metabolome Database [36] and the Phytochemical Interactions Database [37].

The following substances were identified in the Java-type citronella and palmarosa hydroethanolic extracts in a confirmatory manner using standard compounds (Figure S1 and Table S11): *p-*hydroxy benzoic, *o-*hydroxy benzoic, *p-*coumaric, ferulic, caffeic, 3-caffeoyl quinic, and 4-caffeoyl quinic acids and luteolin, luteolin-6*-C-*glucoside, and apigenin-8*-C-*glucoside. *m-*Hydroxy benzoic acid; two isomers of caffeoyl quinic acid; two isomers of coumaric acid and three isomers of feruloyl quinic acid; the flavones apigenin*-C-*hexoside, apigenin*-C-*hexoside*-C-*pentoside, and apigenin-*C,C*-dihexoside; two isomers of luteolin*-C-*hexoside*-C-*pentoside; two isomers of luteolin-*C,C*-dipentoside; luteolin*-O-*desoxyhexosyl*-C-*hexoside; luteolin-*O-*hexoside*-C-*pentoside; luteolin*-O-*rutinoside; and tricin were tentatively (presumptively) identified.

The quantification of compounds in the Java-type citronella and palmarosa extracts was performed using the external calibration method; the data are reported as the mean values ± standard deviations (*n* = 3) (Table S12). The major phenolic components of the hydroalcoholic extracts of both plants were feruloyl quinic acid, luteolin-6*-C-*glucoside, apigenin*-C-*hexoside*-C-*pentoside, luteolin*-C-*hexoside*-C-*pentoside, and tricin. 

### 2.5. Antioxidant Activity of Cymbopogon sp. Hydroalcoholic Extracts 

The antioxidant activities, expressed as μmol Trolox^®^/g extract, determined for the *Cymbopogon* sp. extracts under study, using the ORAC and ABTS^+●^ assays, are presented in Table 4.

## 3. Discussion

The biosynthesis of plant secondary metabolites, such as terpenes and phenolic compounds, is influenced by both biotic and abiotic factors [38,39], including environmental conditions, the use of fertilizers, phytosanitary control, and the phenological stage of the plants, among other factors.

Analysis of variance of the yields of the EOs distilled through MWHD or S.D. indicated that the origin (five different municipalities) had no significant effect on the citronella (Table S1). The yields of palmarosa EOs distilled through MWHD or S.D. showed significant differences according to the origin of the plants; the yields of EOs distilled in Bucaramanga were lower compared with those distilled in the other municipalities (Table S1). Other authors [15,40,41,42,43] have reported EO yields similar to those found in this study (0.8–1.5% for Java-type citronella EO and 0.3–0.6% for palmarosa EO). However, the annual production of EOs calculated for Java-type citronella (154.9 kg EO per ha^−1^ year^−1^) and palmarosa (70.4 kg EO per ha^−1^ year^−1^) cultivated in the experimental plots were much lower than the production levels reported from India (between 387 and 454 kg of palmarosa oil per ha^−1^ year^−1^) [41] and the Philippines (338 kg of citronella oil per ha^−1^ year^−1^) [42], which could be mainly due to the low plant densities employed in these experimental plots (5000 citronella plants per ha^−1^ and 3000 palmarosa plants per ha^−1^), plus other differences in environmental and crop conditions (plantation age, fertilization, and irrigation frequency). 

Although the MWHD experimental time was shorter than that of S.D., and although this technique affords similar yields, the operation scale of available MWHD instrumentation remains in the order of 1 kg of plant material. S.D. is a well-established technique for productive operation with batches of several hundred kilograms of plant material. Apart from differences in the initial investment in instrumentation, MWHD operational costs may be proportionally higher than those of S.D. because of its dependence on electricity to generate steam. S.D. has the advantage of flexibility in this regard because it may employ natural gas or various kinds of fuel obtained from vegetal waste to generate steam at lower costs.

**Figure 3:**
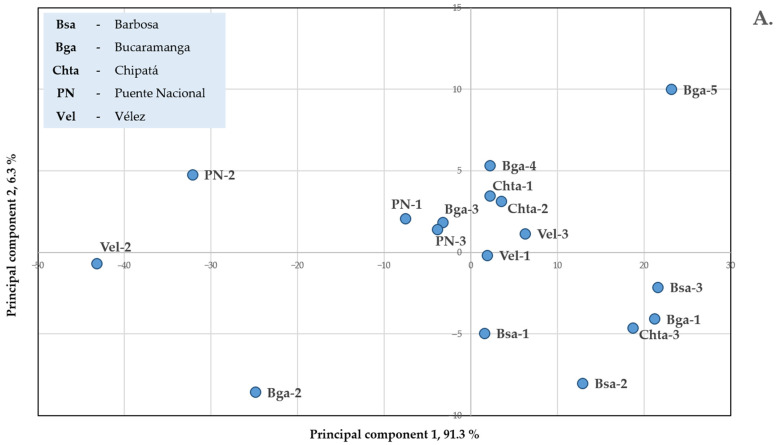

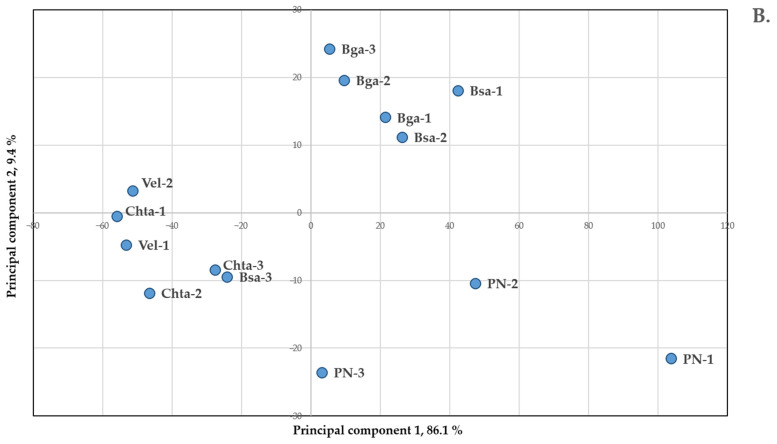
Principal component score plots for chemical compositions of the EOs from (**A**) *C. martinii* and (**B**) *C. winterianus* plants harvested from different municipalities of Santander, Colombia.

Analysis of variance showed that the chemical compositions of the EOs were significantly influenced by the cultivation site of both plant species (Tables S4–S6). This was manifested in the principal component analysis (PCA) results presented in Figure 3. Palmarosa EOs from different harvests from the five municipalities are represented in the score plot of the first two principal components (PC, Figure 3A), which summarizes over 97% of the compositional variance. The main contributor to the first principal component was geraniol (97.8%). For the second principal component, it was geranyl acetate (97.7%). Variable loadings are presented in Table S16. Palmarosa EOs from Barbosa occupied a single quadrant with positive PC1 coordinates and negative PC2 coordinates, while those from Puente Nacional were found in the opposite quadrant with negative PC1 coordinates and positive PC2 coordinates. Palmarosa oils from Chipatá had positive PC1 coordinates, and oils from the remaining locations showed no tendency. Similarly, the PCA of the *C*. *winterianus* EO composition data showed that over 95% of the information can be represented along the first two principal components. The corresponding score plot does contain a more noticeable clustering of EOs according to plantation location. The Java citronella EOs from Bucaramanga and Puente Nacional appear as separate groups, while the oils from the remaining locations occupy a common region. Citronellal was the main contributor (87%) to the definition of PC1, while citronellol (70%) and geraniol (19%) were the main constituents of PC2 (see Table S17).

Citronellal (34–43%) was the main component of the EOs from Java-type citronella cultivated in the municipalities of Santander, followed by geraniol (20–24%), citronellol (10–19%), and geranyl acetate (1–5%). The citronellol amount was, in some cases, slightly higher than that recommended by ISO 3848:2016 (8.5–13%) [16]. The Java-type citronella EO chemical composition determined in this study agreed with those reported by other authors [5,44]. The repellent and larvicidal activities of germacrene D-4-ol, a sesquiterpenol present in the citronella EOs under study (3.26 ± 0.7%), have been previously reported [45,46]. This sesquiterpenol has also been detected in EOs distilled from citronella grown in Brazil, but it was found in much lower concentrations (0.64%) [5].

The main components of the palmarosa EOs studied were geraniol (84 ± 1%), geranyl acetate (5 ± 1%), and linalool (3.0 ± 0.7%); the chemical composition of the palmarosa EO distilled in this study was close to that recommended by ISO 4727:2021 [17]. The relative amounts of geraniol in the EOs of palmarosa cultivated in municipalities of Santander (Colombia) were higher than those reported by Rajeswara et al. [47], Scherer et al. [48], and Devi et al. [49] for palmarosa grown in India. Geraniol has been extensively studied for its antimicrobial, fungicidal, insecticidal, and antitumoral activities [50,51]. It is also used as a natural ingredient of great importance in perfume manufacturing. 

The physicochemical properties measured for Java-type citronella and palmarosa EOs obtained through S.D. from plant material grown in different municipalities of Santander were consistent with the values recommended in the ISO 3848:2016 [16] and ISO 4727:2021 [17] standards, respectively. 

The citronella and palmarosa biomass that remains after distillation (98%) is used for fertilizer, biofuel, construction materials, and paper, but it can be also subjected to prior extraction to isolate substances of biological interest. Citronella and palmarosa extracts were obtained from the residual biomass of plants collected from three harvests (Tables S9 and S10). Their chemical compositions and antioxidant activities were compared with those of extracts isolated from fresh plant material, prior to distillation, to determine if there were variations in the chemical compositions or antioxidant activities caused by the thermal decomposition of the compounds during distillation (Table S13). Extraction yields (11 ± 3% for citronella and 4 ± 1% for palmarosa, Table S9) were similar to those reported by Nurain et al. (3.10%) [52] and Clain et al. (8.72%) [53] for other species of the genus *Cymbopogon*. 

Mainly *C*-glycosylated flavones and hydroxycinnamic acids (30 compounds) were identified using LC/MS on the citronella and palmarosa hydroalcoholic extracts. The mass spectrum of apigenin-*C,C*-dihexoside is shown in Figure S3 as an example of the typical breakdowns and cleavages that *C*-glycosylated flavones undergo during their fragmentation [54]. In the mass spectra of the *C*-glycosylated and *C*-diglycosylated flavones, tentatively identified in the extracts under study, the typical product ions of the cleavage of the C-C bonds that join the sugars with the aglycones were registered, corresponding to losses of C_2_H_4_O_2_ (60 Da), C_4_H_8_O_4_ (120 Da), and C_5_H_10_O_5_ (150 Da) from the protonated molecules [M + H]^+^ or from the product ions [(M + H) − H_2_O]^+^, [(M + H) − 2H_2_O]^+^, and [(M + H) − 3H_2_O]^+^. 

In the mass spectrum of apigenin-8*-C-*glucoside, whose identity was confirmed using the standard substance, the typical product ions [(M + H) − H_2_O]^+^ at *m*/*z* 415.10211 (36%), [(M + H) − 2H_2_O]^+^ at *m*/*z* 397.09137 (20%), and [(M + H) − 3H_2_O]^+^ at *m*/*z* 379.08115 (4%) appeared. Their intensities were lower than those registered for these ions in the mass spectrum of the apigenin*-C-*hexoside isomer (*m*/*z* 415.0172 (57%), *m*/*z* 397.09109 (69%) and *m*/*z* 379.08060 (43%)). Some authors [55,56] found that the sugar attached to the C-6 position of apigenin causes the removal of a water molecule because of the proximity of the hydroxyl groups of the sugar and those of the aglycone at the C-5 and C-7 positions. Therefore, compound N° 26 (Figure 2) was probably apigenin-6*-C-*hexoside. 

The chemical compositions of hydroalcoholic extracts from different plants of the *Cymbopogon* genus have been described in several works [27,29,57,58,59], but to the best of our knowledge, there are no previous studies on the chemical compositions of citronella (C. *winterianus*) and palmarosa (C. *martinii*) extracts. The analysis of lemongrass (*C*. *citratus*) extracts [60,61] found compounds in common, such as 3-caffeoyl quinic (25.2 mg/100 g dry plant), caffeic (2.94 mg/100 g plant), 4-caffeoyl quinic (3 mg/100 g plant), and 5-caffeoyl quinic (44.9 mg/100 g dry plant) acids, although the amounts found in the plants studied here were higher (Table S11). A decrease in flavonoid contents was noticed in the extracts obtained from the residual biomass of citronella and palmarosa in comparison with their amount in the extracts isolated from fresh (undistilled) plants, possibly because of the degradation of some compounds during the distillation process or their solubility in water remaining in the still (Table S12). 

The residual biomass of citronella and palmarosa was used to obtain hydroalcoholic extracts, which were composed mainly of C-glycosyl flavones and hydroxy cinnamic and caffeoyl quinic acids. These are natural substances of interest thanks to their biological activities, as reported in the literature: antihyperglycemic [62], antispasmodic [63], antidiabetic [62], antioxidant [64], anti-inflammatory [65], antinociceptive [66], and anxiolytic [67].

The antioxidant activities (measured using the ORAC and ABTS^+●^ assays) of the citronella and palmarosa hydroalcoholic extracts obtained from fresh or waste plant material were very similar (Table S15) but one order of magnitude lower than the values measured for some of their individual components (Table 4) and those reported for extracts from other *Cymbopogon* species. Nurain et al. [52] reported an antioxidant activity value of 8880 ± 1 μmol Trolox^®^/g dry extract for fresh *C*. *citratus* extracted with a 95% ethanolic solution. Clain et al. [53] evaluated the antioxidant activity of *C*. *nardus* (Ceylon-type citronella) extracts resulting from different extraction methods. These authors reported an antioxidant activity of 1744 ± 155 μmol Trolox^®^/g extract for the Soxhlet hydroethanolic extract.

This first study on the chemical composition and antioxidant activities of extracts obtained from the residual biomass of citronella and palmarosa distillation showed that postdistillation waste could eventually serve as a natural source of bioactive substances. Approximately 50 mg was found per gram of hydroxybenzoic acid and flavone dry extract. Since the extract composition is not dominated by a single substance, the separation of individual constituents is a complex and expensive process. Therefore, it is more practical to develop applications that take advantage of these extracts as mixtures of bioactive molecules that exhibit a combination of interesting chemical and biological reactivities. The comprehensive utilization of these *Cymbopogon* species includes the production of essential oils, hydrosols, and extracts and the final utilization of the exhausted plant material in composting, biochar, or biofuel generation. 

## 4. Materials and Methods

### 4.1. Chemical Substances and Reagents

The standard substances β-myrcene (94%), linalool (97%), geraniol (98%), geranial (95%), geranyl acetate (98%), limonene (97%), (*E*)-β-caryophyllene (98.5%), citronellal (90%), isopulegol (99%), citronellol (95%), eugenol (99%), (2*E*,6*Z*)-farnesol (96%), germacrene D (90%), caffeic acid (>98), ferulic acid (≥99%), *p-*coumaric acid (≥98%), *p-*hydroxy benzoic acid (≥99%), *o-*hydroxy benzoic acid (99.98%), luteolin-6*-C-*glucoside (98%), AAPH (97%), and fluorescein (99%) were purchased from Sigma-Aldrich (St. Louis, MO, USA); kaempferol-3*-O-*rutinoside (98%), and luteolin-7*-O-*glucoside were purchased from ChemFaces (Wuhan, China); apigenin-8*-C-*glucoside (≥95%), 3-caffeoyl quinic acid (≥99%) and 4-caffeoyl quinic acid (≥98%) were obtained from PhytoLab GmbH (Vestenbergsgreuth, Bavaria, Germany); and luteolin (≥95%) was obtained from Santa Cruz Biotechnology (Dalas, TX, USA) GC/MS-grade dichloromethane, LC/MS-grade methanol, combi-Titran 5, hydrochloric acid (37%), ammonium formate (99.8%), formic acid (99.8%), potassium persulfate (99%), and absolute ethanol (96%), were purchased from Merck (Darmstadt, Germany). ABTS (2,2′-azino-bis(3-ethylbenzothiazoline-6-sulfonic) acid) and Trolox^®^ (97%) were purchased from Sigma-Aldrich (St. Louis, MO, USA), ethanol (96%) was purchased from SUQUIN (Bucaramanga, Santander, Colombia), and propylene glycol (99.9%) was obtained from Biopharm Inc. (Hatfield, AR, USA). 

### 4.2. Vegetal Material

Citronella and palmarosa plants were harvested from experimental plots in the municipalities of Barbosa (5°57′5.083″ N 73°37′32.146″ W), Chipatá (6°3′24.998″ N 73 °37′40.998″ W), Puente Nacional (5°51′55.058″ N 73°39′50.378″ W), Vélez (6°0′14.198″ N 73°37′37.650″ W), and Bucaramanga (7°8′26.376″ N 73°6′57.851″ W), Santander, Colombia, in 2021 and 2022.

The crops of each species were established in an area of 3400 m^2^, with a planting density of 2 citronella plants per m^−2^ and 3.3 palmarosa plants per m^−2^, distributed in ten plots in each region for a total of 1.4 ha. The first formative pruning was performed four months after planting. The first citronella harvest was carried out four months after the formative pruning. Palmarosa plants were harvested after flowering and before seed formation. 

### 4.3. Essential Oil Distillation 

#### 4.3.1. Microwave Radiation-Assisted Hydrodistillation 

Hydrodistillation was carried out using Clevenger-type equipment, with a Dean-Stark distillation reservoir (Glass Lab, Bogotá, Cundinamarca, Colombia) adapted to a Samsung domestic microwave oven, model AME1114TST, with an output power of 1.6 kW and a radiation frequency of 2450 MHz. Oven power (600 W) was set to 60% of its maximum value. Plant material (450 g) and water (ca. 300 mL) were placed in a glass distillation flask (2 L). The total distillation time was 45 min, divided into three intervals of 15 min each, with a rest time of 5 min. The EOs distilled were filtered and dried with anhydrous Na_2_SO_4_ prior to their chromatographic analysis.

#### 4.3.2. Steam Distillation

The EOs from the chopped plant material (leaves and stems) of palmarosa or citronella were obtained through steam distillation (S.D.) in a 1 m^3^ stainless steel still, coupled with a tubes-and-casing stainless steel condenser (INAL, Bucaramanga, Colombia) using water from a cooling tower (Glaciar, La Estrella, Colombia). Steam was generated in a 6 BHP gas-fired boiler (Tecnik, Bogotá, Colombia) operated at 80 psi (5 × 10^5^ Pa). The plant material in the still was compacted to a density of 250 and 300 kg m^−3^. Typically, S.D. with a condensate flow of 740 mL min^−1^ lasted for ca. 2 h. The separation of EO and hydrolate was carried out via differences in densities in a Florentine stainless-steel vessel (INAL, Bucaramanga, Colombia). The EO was filtered and dried with anhydrous sodium sulfate. All distillations were carried out in triplicate.

### 4.4. Solvent Extraction

Extraction with hydroethanolic solution (70% *v*/*v*) was carried out following the methodology described by Rodrigues et al. [68], with some modifications. The plant material (100 g) before the distillation (fresh) or the residual biomass, dried and ground, was mixed with an aqueous ethanol solution (2 L, 70% *v*/*v*) and deposited in an ultrasonic bath (Elmasonic S15H, Singen, Germany) at 50 °C for 1 h. The extracts were filtered and concentrated in a Heidolph rotary evaporator (Hei-VAP, Advantage HL, Chicago, IL, USA) and then lyophilized (VirTis AdVantage Plus, SP Scientific, Gardiner, NY, USA) and stored at 4 °C, protected from light. Solvent extractions were performed in triplicate. 

### 4.5. Essential Oil Physicochemical Properties

Physicochemical properties were measured in triplicate to determine the quality of the EOs obtained through S.D. from plants grown in Puente Nacional, Chipatá, Vélez, Barbosa, and Bucaramanga (Santander, Colombia), following ISO standards. Relative density at 20 °C (ISO 279:1998) [69], miscibility in ethanol (ISO 875:1999) [70], optical rotation (ISO 592:1998) [71], boiling point (ISO/TR 11018:1997) [72], water content (ISO 11021:1999) [73], freezing point (ISO 1041:1973) [74], refractive index (ISO 280:1998) [75], acid value (ISO 1242:1999) [76], and ester value (ISO 709:2001) [77] were determined. 

### 4.6. GC/FID/MS Analysis 

A 6890 Plus gas chromatograph (Agilent Technologies, AT, Palo Alto, CA, USA) equipped with a mass selective detector MS 5973 Network (AT, Palo Alto, CA, USA) using electron ionization (EI, 70 eV) was employed for EO analysis. Compound separation was carried out on a DB-5MS nonpolar column (J & W Scientific, Folsom, CA, USA) (5%-phenyl-poly(methylsiloxane), 60 m × 0.25 mm (I.D.) × 0.25 (d_f_)) and on a DB-WAX polar column (J & W Scientific, Folsom, CA, USA) (poly(ethylene glycol), 60 m × 0.25 mm (I.D.) × 0.25 (d_f_)). Helium (99.995%, AP gas, Messer, Bogotá, Colombia) was used as carrier gas at a column inlet pressure of 113.5 kPa and a constant flow of 1 mL min^−1^. Samples were injected into the GC/MS (1 µL) in split mode (1:30). The injector, ionization chamber, and quadrupole temperatures were maintained at 250, 230, and 150 °C, respectively. The mass range examined was *m*/*z* 45–450 (3.58 scans/s). Compound quantification was performed using the external standard calibration method on an AT 6890N gas chromatograph (AT, Palo Alto, CA, USA) coupled with a flame ionization detector (FID) operated at 250 °C with gas flows of air (300 mL/min), hydrogen (30 mL/min), and nitrogen (make-up) (30 mL/min). The GC/MS and GC/FID data were processed with MSDChemStation G1701DA (AT, Palo Alto, CA, USA) and ChemStation version B.04.03-SP1, respectively.

The tentative identification of the compounds was based on the comparison of the linear retention indices (LRIs) measured on the nonpolar (DB-5MS) and polar (DB-WAX) columns, and fragmentation patterns from the literature and the spectral databases of Adams (2007) [33], NIST (2017) [34], and Wiley (2008) [35]. For confirmatory identification, the following standard compounds were used: β-myrcene, linalool, nerol, geraniol, geranial, geranyl acetate, (*E*)-β-caryophyllene, (2*E*,6*Z*)-farnesol, limonene, citronellal, *iso*-pulegol, citronellol, and eugenol. 

### 4.7. UHPLC-ESI-Orbitrap-HRMS Analysis

The hydroethanolic extracts of C. *martinii* (palmarosa) and C. *winterianus* (citronella) were analyzed in an ultra-high-performance liquid chromatograph (UHPLC) VanquishTM (Thermo Scientific, Waltham, MA, USA) coupled with an Exactive Plus Orbitrap (Thermo Scientific, Bremen, Germany) mass spectrometer with a heated electrospray interface (HESI), operated in dual positive and negative ion acquisition mode at 350 °C with a capillary voltage of 3.5 kV. Nitrogen (>99%) produced by an NM32LA generator (Peak Scientific, Scotland, U.K.) was used as the drying and nebulizing gas.

Separation of compounds in extracts was performed on a ZORBAX Eclipse XDB-C_18_ column (Sigma-Aldrich, St. Louis, MO, USA), 50 mm, L × 2.1 mm, I.D. × 1.8 μm particle size, at 40 °C. The mobile phase was A—water (0.1% formic acid, FA; 5 mM ammonium formate, AF) and B—methanol (0.1% FA and 5 mM AF). The mobile phase gradient started at 100% A and changed linearly to 100% B in eight minutes, held constant for four minutes, then returned to 100% A in one minute, and equilibrated for seven minutes. The mobile phase flow was 0.3 mL min^−1^, and the injection volume was 2 μL.

The Orbitrap-MS operated in full scan mode with a resolution of 70,000 (full-width-at-half-maximum, RFWHM, *m*/*z* 200), an automatic gain control (AGC) of 1 × 10^6^, and an injection time in the C-Trap of 200 ms. The HCD chamber was used in the step-scan acquisition mode for each collision energy (10–70 eV), with RFWHM = 35,000 (*m*/*z* 200), AGC= 2 × 10^5^, and a 100 ms injection time. The mass range was recorded in the *m*/*z* 80–1000 mass range. 

Data were processed with the Thermo XcaliburTM Roadmap software, Version 3.1.66.10. Compound identification was based on the EICs of the target analytes using metabolomics (HMDB) [36] and phytochemistry (PCIDB) [37] databases and the standard substances of *p-*hydroxy benzoic, *p-*coumaric, ferulic, caffeic, 3-caffeoyl quinic, 4-caffeoyl quinic acids, luteolin, luteolin-6*-C-*glucoside, and apigenin-8*-C-*glucoside. 

### 4.8. Antioxidant Activity 

#### 4.8.1. ABTS^+●^ Radical-Cation Decoloration Assay 

ABTS^+•^ radical-cation decolorization assays were performed using a Modulus^®^ II microplate reader (Turner Biosystems Inc., Sunnyvale, CA, USA) at λ = 750 nm. The ABTS^+•^ solution was obtained from a mixture of ABTS (7 mM in sodium acetate buffer (CH_3_COONa), pH 4.5) and potassium persulfate (2.45 mM), prepared using sonication for 30 min; the mixture was stored at 4 °C for 24 h in the absence of light to obtain a stable solution. The ABTS^+•^ solution was then diluted in sodium acetate buffer until an absorbance of 0.71 ± 0.02 was obtained; the mixture was stored at 4 °C (30 min) before use. The extracts were weighed, dissolved in absolute ethanol, and diluted in sodium acetate buffer (20 mM, pH 4.5). The diluted extract (10 µL) and the ABTS^+•^ solution (190 µL) were deposited in each well of the plate; absorbance was measured for 60 min at 25 °C. The calibration curve was carried out using Trolox^®^ as a reference substance; the measurements were performed in triplicate, and the antioxidant capacity values were expressed (μmol Trolox^®^/g of dry extract) as mean values ± standard deviations. 

#### 4.8.2. Evaluation of Oxygen-Radical Absorption Capacity (ORAC)

Oxygen-radical absorbance capacity measurements were performed with a Modulus^®^ II microplate reader (Turner Biosystems Inc., Sunnyvale, CA, USA). The extract was weighed and dissolved in methanol (HPLC grade); then, dilutions were made in phosphate buffer (15 mg/L). The diluted sample was deposited in each reading plate well (25 μL) with a fluorescein solution (150 μL, 81 nM in phosphate buffer); the mixture was incubated at 37 °C (20 min), and then, a solution of AAPH (25 μL, 153 mM, in phosphate buffer) was added. Fluorescence was measured (37 °C, 90 min) using an excitation wavelength of λ = 490 nm and an emission wavelength of λ = 510 nm. Antioxidant protection was determined based on the difference between the area under the fluorescence curve (AUC) obtained for each sample and for the blank (25 μL of phosphate buffer). Trolox^®^ was used as the reference substance. All measurements were carried out in triplicate.

### 4.9. Data Analysis

Analysis of variance (ANOVA) was used to determine the significance of plant material origin on the yields and chemical compositions of EOs from Java-type citronella and palmarosa plants. Results from yields, chemical compositions, and antioxidant assays were analyzed using ANOVA to establish significant differences (*p <* 0.05) from the hydroalcoholic extracts of Java-type citronella and palmarosa isolated from plant material prior to distillation and postdistillation waste. Principal component analysis was performed with version 3.1 of the CAPCA add-in for Microsoft Excel.

## 5. Conclusions

The palmarosa and citronella EOs were distilled from plants grown in five municipalities (Barbosa, Bucaramanga, Chipatá, Puente Nacional, and Vélez) of Santander, Colombia. The citronella EOs were characterized by high citronellal (34–43%) and citronellol (11–19%) content, while the palmarosa EOs contained geraniol (83–87%), a compound recognized for its insecticidal, repellent, and antifungal activities. The values of the physicochemical properties of the EOs corresponded to those recommended by ISO standards. The residual biomass was used to obtain hydroalcoholic extracts that turned out to be rich in *C*-glycosylated flavones and hydroxycinnamic acids, compounds of interest for their antihyperglycemic and antispasmodic activities. Tricin (8 ± 2 mg g^−1^ extract) and apigenin*-C-*hexoside*-C-*pentoside (6.65 ± 0.07 mg g^−1^ extract) were major compounds in the extract of palmarosa, and luteolin-6*-C-*glucoside (6.0 ± 0.5 mg g^−1^ extract), luteolin*-O-*desoxyhexosyl*-C-*hexoside (5.9 ± 0.1 mg g^−1^ extract), and luteolin*-C-*hexoside*-C-*pentoside (5.1 ± 0.2 mg g^−1^ extract) prevailed in the citronella extract (Table S12). From residual palmarosa and citronella residual biomass, up to 3 kg of tricin per ha^−1^ year^−1^ and 0.6 kg of luteolin-6*-C-*glucoside per ha^−1^ year^−1^ could be obtained, respectively. The results of this research show that the EOs distilled from citronella and palmarosa plants grown in Santander, Colombia, have compositions and physicochemical characteristics suitable for their commercialization in the international market. As an approach to integral and sustainable use, the residual biomass after the distillation of *C*. *winterianus* and *C*. *martinii* can, among other uses, obtain extracts that could be used directly or as sources of natural ingredients for different final products of the cosmetic, pharmaceutical, and cleaning industries.

## Figures and Tables

**Table 1:** Yields of essential oils, distilled via S.D. and MWHD, from Java-type citronella and palmarosa harvested from different municipalities of Santander, Colombia.

Collection Place	Number of Harvests, *n*	Yield, % ± SD			
		S.D.		MWHD	
		Citronella	Palmarosa	Citronella	Palmarosa
Barbosa	3	0.7 ± 0.1	0.37 ± 0.09	1.0 ± 0.2	0.4 ± 0.1
Bucaramanga	5	0.7 ± 0.1	0.26 ± 0.05	0.7 ± 0.2	0.32 ± 0.07
Chipatá	3	0.8 ± 0.1	0.37 ± 0.08	0.9 ± 0.2	0.5 ± 0.3
Puente Nacional	3	0.9 ± 0.1	0.44 ± 0.07	1.0 ± 0.4	0.42 ± 0.09
Vélez	2–3 *	0.9 ± 0.1	0.40 ± 0.08	1.2 ± 0.1	0.4 ± 0.1
Average yield, %		0.8 ± 0.1	0.37 ± 0.06	1.0 ± 0.2	0.4 ± 0.1

S.D.: steam distillation. MWHD: microwave-assisted hydrodistillation. * Citronella (*n* = 2) and palmarosa (*n* = 3).

**Table 2:** Chemical composition of the EOs, distilled via MWHD, from Java-type citronella plants harvested from different municipalities of Santander (Colombia).

N° Figure 1A	Compound	Linear Retention Indices						GC/FID Relative Peak Area, % ± SD						
		DB-5MS			DB-WAX			ISO 3848:2016 [16]		Municipalities				
		Exp	Std	Lit	Exp	Std	Lit	Min.	Max.	Barbosa (*n* = 3)	Bucaramanga (*n =* 5)	Chipatá (*n* = 3)	Puente Nacional (*n* = 3)	Vélez (*n* = 2)
1	Limonene ^a,b,c^	1032	1035	1030 [30]	1202	1203	1198 [30]	2.0	5.0	3.1 ± 0.3	2.21 ± 0.07	2.7 ± 0.2	2.6 ± 0.3	3.2 ± 0.3
2	Linalool ^a,b,c^	1101	1102	1099 [30]	1552	1552	1543 [30]	0.5	1.5	0.77 ± 0.06	0.43 ± 0.01	0.7 ± 0.1	0.87 ± 0.06	0.8 ± 0.1
3	Citronellal ^a,b,c^	1158	1157	1154 [30]	1491	1486	1475 [30]	31	39	41 ± 4	43.0 ± 0.3	34 ± 1	37 ± 3	41 ± 1
4	*iso*-Pulegol ^a,b,c^	1165	1157	1156 [31]	1576	1584	1566 [32]	0.5	1.7	0.17 ± 0.06	0.135 ± 0.002	0.17 ± 0.06	0.20 ± 0.01	0.20 ± 0.01
5	Citronellol ^a,b,c^	1229	1230	1228 [30]	1769	1769	1763 [30]	8.5	13	14 ± 3	10.72 ± 0.05	16 ± 2	19 ± 2	15 ± 2
6	Neral ^a,b^	1241	-	1242 [30]	1692	-	1679 [30]			0.6 ± 0.1	0.596 ± 0.002	0.7 ± 0.1	0.4 ± 0.1	0.85 ± 0.07
7	Geraniol ^a,b,c^	1255	1255	1255 [30]	1857	1855	1839 [30]	20	25	24.4 ± 0.6	19.97 ± 0.08	24 ± 1	23 ± 4	23.5 ± 0.3
8	Geranial ^a,b,c^	1270	1272	1270 [30]	1740	1736	1725 [30]	0.3	11	0.9 ± 0.1	0.863 ± 0.002	1.0 ± 0.1	0.7 ± 0.1	1.2 ± 0.1
9	Citronellyl acetate ^a,b^	1345	-	1352 [30]	1666	-	1656 [30]	2.0	4.0	2.0 ± 0.2	1.33 ± 0.01	3.3 ± 0.1	2.0 ± 0.2	2.3 ± 0.1
10	Eugenol ^a,b,c^	1353	1361	1358 [30]	2184	2167	2163 [30]	0.5	1.0	1.1 ± 0.1	1.014 ± 0.003	1.2 ± 0.1	0.9 ± 0.2	1.0 ± 0.1
11	Geranyl acetate ^a,b,c^	1374	1387	1380 [30]	1760	1759	1751 [30]	2.5	5.5	2.5 ± 0.6	1.76 ± 0.01	4.7 ± 0.9	1.9 ± 0.2	2.9 ± 0.4
12	β-Elemene ^a,b^	1394	-	1390 [30]	1599	-	1591 [30]	0.7	2.5	0.5 ± 0.1	0.98 ± 0.02	0.63 ± 0.06	0.57 ± 0.06	0.5 ± 0.1
13	Germacrene D ^a,b^	1490	-	1481 [30]	1719	-	1708 [30]	1.5	3.0	1.2 ± 0.2	1.95 ± 0.01	1.5 ± 0.1	1.5 ± 0.1	1.25 ± 0.07
14	δ-Cadinene ^a,b^	1525	-	1523 [30]	1764	-	1756 [30]	1.4	2.5	0.5 ± 0.1	1.18 ± 0.01	1.1 ± 0.2	0.3 ± 0.1	0.7 ± 0.1
15	Elemol ^a,b^	1557	-	1548 [30]	2086	-	2079 [30]	1.3	4.0	2.0 ± 0.4	4.7 ± 0.1	2.3 ± 0.6	1.7 ± 0.3	1.3 ± 0.1
16	Germacrene D-4-ol ^a,b^	1587	-	1574 [30]	2059	-	2057 [30]			3 ± 1	4.0 ± 0.1	3.1 ± 0.9	3.9 ± 0.8	2.3 ± 0.1
17	α-Cadinol ^a,b^	1666	-	1652 [30]	2243	-	2227 [30]			0.3 ± 0.1	1.59 ± 0.02	0.6 ± 0.2	0.2 ± 0.1	0.45 ± 0.07
Total GC peak area, %										97.87	96.49	97.53	97.54	99.25

Exp: Experimental values. Lit: values reported in the literature [30,31,32]. Std: values determined for the standard compounds used in this study. ^a^ Tentative identification based on linear retention indices (LRIs), measured on DB-5MS (nonpolar) and DB-WAX (polar) columns [30]. ^b^ Tentative identification based on mass spectrum (MS; EI, 70 eV, coincidence > 95%) study of fragmentation patterns (EI, 70 eV) and comparison with mass spectra from Adams (2007) [33], NIST (2017) [34], and Wiley (2008) [35] databases. ^c^ Confirmatory identification based on the standard substances (Std) limonene (97%), linalool (97%), citronellal (90%), *iso*-pulegol (99%), citronellol (95%), geraniol (98%), geranial (95%), eugenol (99%), and geranyl acetate (98%) and the comparison of their mass spectra (MS; EI, 70 eV) and LRIs with those of the compounds detected in the EOs studied.

**Table 3:** Chemical composition of the EOs, distilled via MWHD, from palmarosa plants harvested from different municipalities of Santander (Colombia).

N° Figure 1B	Compound	Linear Retention Indices						GC/FID Relative Peak Area, % Mean ± SD						
		DB-5MS			DB-WAX			ISO 4727:2021 [17]		Municipalities				
		Exp	Std	Lit	Exp	Std	Lit	Min.	Max.	Barbosa (*n =* 3)	Bucaramanga (*n =* 5)	Chipatá (*n =* 3)	Puente Nacional (*n* = 3)	Vélez (*n* = 3)
1	β-Myrcene ^a,b,c^	990	989	989 [30]	1165	1165	1161 [30]	0.1	0.5	0.23 ± 0.06	0.140 ± 0.003	0.73 ± 0.06	0.3 ± 0.1	0.3 ± 0.1
2	(*Z*)*-*β*-*Ocimene ^a,b^	1037	-	1038 [30]	1237	-	1235 [30]			0.3 ± 0.1	0.243 ± 0.002	0.3 ± 0.1	0.3 ± 0.1	0.3 ± 0.1
3	(*E*)*-*β*-*Ocimene ^a,b^	1048	-	1048 [30]	1254	-	1250 [30]	0.2	2.0	1.4 ± 0.6	1.122 ± 0.007	1.6 ± 0.1	1.4 ± 0.2	1.5 ± 0.3
4	Linalool ^a,b,c^	1101	1102	1099 [30]	1550	1551	1543 [30]	1.5	4.0	3.1 ± 0.8	1.720 ± 0.004	3.9 ± 0.5	3.1 ± 0.4	3.2 ± 0.3
5	Nerol ^a,b,c^	1232	1230	1229 [30]	1806	1806	1795 [30]	0.2	1.0	0.2 ± 0.1	0.13 ± 0.03	0.37 ± 0.06	0.43 ± 0.06	0.43 ± 0.06
6	Neral ^a,b^	1241	-	1242 [30]	1688	-	1679 [30]	0.05	0.3	0.23 ± 0.06	0.163 ± 0.001	0.27 ± 0.06	0.2 ± 0.1	0.2 ± 0.1
7	Geraniol ^a,b,c^	1259	1253	1255 [30]	1858	1853	1839 [30]	77	85	84 ± 2	84.4 ± 0.1	83 ± 3	87.5 ± 0.2	83 ± 3
8	Geranial ^a,b,c^	1272	1271	1270 [30]	1735	1735	1725 [30]	0.1	0.6	0.7 ± 0.2	0.60 ± 0.04	0.8 ± 0.2	0.47 ± 0.06	0.6 ± 0.2
9	Geranyl acetate ^a,b,c^	1377	1378	1380 [30]	1756	1758	1751 [30]	5	13	7 ± 1	5.50 ± 0.04	4 ± 1	4.9 ± 0.3	5 ± 1
10	(*E*)-β-Caryophyllene ^a,b,c^	1434	1434	1420 [30]	1609	1611	1599 [30]	1	2.5	0.5 ± 0.1	0.763 ± 0.002	0.7 ± 0.1	0.37 ± 0.06	0.43 ± 0.06
11	(*2E,6Z-*)*-*Farnesol ^a,b,c^	1718	1718	1714 [30]	2360	2361	2359 [30]	*tr*	1.5	0.4 ± 0.1	1.60 ± 0.02	0.5 ± 0.1	0.3 ± 0.1	0.3 ± 0.1
Total GC peak area, %										97.87	96.49	97.53	97.54	99.25

Exp: experimental values. Lit: values reported in the literature [30]. Std: values determined for the standard compounds used in this study. *tr:* traces. ^a^ Tentative identification based on linear retention indices (LRIs) measured on DB-5MS (non-polar) and DB-WAX (polar) columns [30]. ^b^ Tentative identification based on mass spectrum (MS; EI, 70 eV, coincidence > 95%) study of fragmentation patterns (EI, 70 eV) and comparison with mass spectra from Adams (2007) [33], NIST (2017) [34], and Wiley (2008) [35] databases. ^c^ Confirmatory identification based on the standard compounds (Std) β-myrcene (94%), linalool (97%), nerol (97%), geraniol (98%), geranial (95%), geranyl acetate (98%), (*E*)-β-caryophyllene (98.5%), and (*2E*,*6Z*)-farnesol (96%) and the comparison of their mass spectra (MS; EI, 70 eV) and LRIs with those of the compounds detected in the EOs studied.

**Table 4:** Antioxidant activities of *Cymbopogon* sp. hydroalcoholic extracts and of individual components.

Extracts/Compounds	Vegetal Material	µmol Trolox^®^/g Extract, Mean ± SD (*n* = 3)	
		ORAC	ABTS^+●^
Java-type citronella	Before distillation	1100 ± 40	71 ± 2
	Postdistillation waste	1400 ± 837	78 ± 1
Palmarosa	Before distillation	1300 ± 61	167 ± 1
	Postdistillation waste	1400 ± 118	103 ± 7
*p-*Coumaric acid		17,600 ± 704	8700 ± 218
Ferulic acid		14,200 ± 167	8500 ± 309
3-Caffeoyl quinic acid		14,000 ± 132	2140 ± 88
4-Caffeoyl quinic acid		9900 ± 600	2080 ± 56
Luteolin		18,000 ± 636	4000 ± 151
Luteolin-6*-C-*glucoside		11,600 ± 240	3300 ± 143
Apigenin-8*-C-*glucoside		10,500 ± 405	*

* There was no response from apigenin-8-*C–*glucoside at the evaluated concentration (550 mg/L).

## Data Availability

The supporting data can be found in the database of the CIBIMOL-CROM-MASS research group, Universidad Industrial de Santander, Bucaramanga, Colombia.

## Supplementary Materials

The following supporting information can be downloaded at https://www.mdpi.com/article/10.3390/molecules28176315/s1: Table S1. Results of the analysis of variance used to evaluate the effect of plant material origin on the yields of essential oils, distilled through MWHD and S.D., from Java-type citronella and palmarosa. Table S2. Results of the analysis of variance used to evaluate the effect of distillation methods, S.D. or MWHD, on the yields of essential oils from Java-type citronella and palmarosa. Table S3. Quantification of the compounds present in Java-type citronella EOs according to plant crop locations. Table S4. Results of the analysis of variance used to evaluate the effect of plant material origin on the chemical composition of the EOs, distilled through MWHD, from Java-type citronella plants. Table S5. Quantification of the compounds present in palmarosa EOs according to plant crop locations. Table S6. Results of the analysis of variance used to evaluate the effect of plant material origin on the chemical composition of the EOs, distilled through MWHD, from palmarosa plants. Table S7. Physicochemical properties of Java-type citronella and palmarosa EOs distilled in Santander compared with ISO 3848:2016 and ISO 4727:2021 data. Table S8. Chemical composition of the EOs, distilled through S.D., from Java-type citronella and palmarosa plants harvested from different municipalities of Santander (Colombia). Table S9. Yields of hydroalcoholic extracts of Java-type citronella and palmarosa plants cultivated in Santander (Colombia). Table S10. Results of the analysis of variance used to evaluate the effect of plant material before distillation or postdistillation waste on yields of hydroalcoholic extracts of Java-type citronella and palmarosa. Table S11. Exact masses of protonated [M + H]^+^ and deprotonated [M − H]^−^ molecules, identified via UHPLC-ESI-Orbitrap-MS, in C. *winterianus* and C. *martinii* hydroalcoholic extracts. Table S12. Quantification of phenolic compounds via UHPLC-ESI-Orbitrap-MS operated in SIM mode, present in the *Cymbopogon* sp. extracts studied. Table S13. Results of the analysis of variance used to evaluate the effect of plant material prior to distillation or biomass waste on the chemical composition of hydroalcoholic extracts of Java-type citronella and palmarosa. Table S14. Detection and quantification limits, obtained via UHPLC-ESI^+/−^-Orbitrap-MS operated in SIM mode, of the standard compounds used in this study. Table S15. Results of the analysis of variance used to evaluate the effect of plant material prior to distillation or biomass waste on the antioxidant activities of hydroalcoholic extracts of Java-type citronella and palmarosa. Table S16. Variable loadings for the principal components of *C. martinii* EO composition data. Table S17. Variable loadings for the principal components of *C. winterianus* EO composition data. Figure S1. Extracted ion currents (EICs), obtained via UHPLC-ESI^+/−^-Orbitrap-MS operated in SIM mode, of the protonated [M + H]^+^ and deprotonated [M − H]^−^ molecules of the substances present in the C. *winterianus* solvent extracts obtained A. prior to distillation plant material and B. from biomass waste. Scale, 6.8 × 10^7^. See peak identification in Table S6. Figure S2. Mass spectrum, obtained via UHPLC-ESI^+^-Orbitrap-MS (HCD, 10 eV), of the deprotonated molecule [M − H]^−^ of compound (C_16_H_18_O_9_) present in Java-type citronella and palmarosa solvent extracts isolated from residual biomass. SIM-EIC of the ion *m*/*z* 353.08777 [M − H]^−^ and the formation of its main products. Figure S3. Mass spectrum, obtained by UHPLC-ESI^+−^Orbitrap-MS (HCD, 20 eV), of apigenin-*C,C*-dihexoside present in Java-type citronella and palmarosa hydroalcoholic extracts isolated from residual biomass. SIM-EIC of the ion at *m*/*z* 595.16498 [M + H]^+^ and the formation of its main products. Figure S4. Geographical location of citronella and palmarosa crops. A. Adapted from Google Maps. B. Palmarosa (*C. martinii*) plantation. C. Citronella (*C. winterianus*) plantation in the experimental plots at the municipality of Barbosa (Santander). 31 August 2021.

## Sample Availability

Samples of Java-type citronella and palmarosa EOs and extracts are available in CENIVAM—CROM-MASS at the Industrial University of Santander (Bucaramanga, Colombia).

## Abbreviations

AAPH	2,2-Azobis-(2-methylpropionamidine) dihydrochloride
ABTS	2,2-azinobis-(3-ethylbenzothiazoline-6-sulfonic acid)-diammonium salt assay
AT	Agilent Technologies
d_f_	Stationary-phase film thickness
I.D.	Column internal diameter
EIC	Extracted ion current
EO(s)	Essential oil(s)
ESI	Electrospray ionization
eV	Electronvolt
FID	Flame ionization detector
GC	Gas chromatography
GC/MS	Gas chromatography coupled to mass spectrometry
HCD	Higher-energy collision dissociation cell
HD	Hydrodistillation
HRMS	High-resolution mass spectrometry
I	Intensity (abundance)
ISO	International Organization for Standardization
LC	Liquid chromatography
LC/MS	Liquid chromatography coupled to mass spectrometry
LOD	Limit of detection
LOQ	Limit of quantification
LRIs	Linear retention indices
MS	Mass spectrometry or mass spectrum (spectra)
MWHD	Microwave-assisted hydrodistillation
*m*/*z*	Mass-to-charge ratio
ORAC	Oxygen-radical absorption capacity assay
S.D.	Steam distillation
SIM	Selected ion monitoring
t_R_	Retention time (min)
UHPLC	Ultra-high-performance liquid chromatography
