# Supplementary material for: Enhanced Two-Step Extraction from Biomass of Two Cymbopogon Species Cultivated in Santander, Colombia
Source: Molecules. 2023 Aug 29;28(17):6315. doi: 10.3390/molecules28176315 (PMC10488661; doi:10.3390/molecules28176315)
Supplement: Supplementary file 1 [file molecules-28-06315-s001.zip › molecules-2523943-supplementary.pdf]

*Supplementary Material*

# Enhanced Two-Step Extraction from Biomass of Two *Cymbopogon* Species Cultivated in Santander, Colombia

**Angie K. Romero [1], Daysy J. Portillo [1], Sheila B. Beltrán [1], Lady J. Sierra [1], Camilo A. Álvarez [2], Karen J. Ramírez [2], Jairo R. Martínez [1,2] and Elena E. Stashenko [1,2,*]**

[1] Research Center for Chromatography and Mass Spectrometry (CROM-MASS), Universidad Industrial de Santander, Bucaramanga 680002, Colombia; cenivam.sgr03@uis.edu.co (A.K.R.); cenivam.sgr01@uis.edu.co (D.J.P.); sheila2218097@correo.uis.edu.co (S.B.B.); lady.sierra2@correo.uis.edu.co (L.J.S.); jmartine@uis.edu.co (J.R.M.)
[2] Research Center for Biomolecules (CIBIMOL), Universidad Industrial de Santander, Bucaramanga 680002, Colombia; camilo.alvarez@uis.edu.co (C.A.Á.); karen2188739@uis.edu.co (K.J.R.)
* Correspondence: elena@tucan.uis.edu.co

**Table S1.** Results of the analysis of variance used to evaluate the effect of plant material origin on the yields of essential oils, distilled by MWHD and S.D., from Java type citronella and palmarosa.

| Essential oils | Distillation method | Yields | | | | | F | *p* |
|---|---|---|---|---|---|---|---|---|
| | | Barbosa | Bucaramanga | Chipatá | Puente Nacional | Vélez | | |
| Citronella | S.D. | A | A | A | A | A | 0.95 | 0.4764 |
| | MWHD | A | A | A | A | A | 1.87 | 0.1993 |
| Palmarosa | S.D. | B | A | B | B | B | 6.02 | 0.0002 |
| | MWHD | A,B | A | A,B | B | A,B | 3.77 | 0.0405 |

S.D.: Steam distillation. MWHD: Microwave-assisted hydrodistillation. A $p$ value <0.05 indicates that the effect is significant with a 95% confidence level (F-test).

**Table S2.** Results of the analysis of variance used to evaluate the effect of distillation methods, S.D. or MWHD, on the yields of essential oils from Java type citronella and palmarosa.

| Essential oils | Distillation methods | | F | *p* |
|---|---|---|---|---|
| | S.D. | MWHD | | |
| Citronella | A | A | 2.98 | 0.1228 |
| Palmarosa | A | A | 0.93 | 0.3628 |

S.D.: Steam distillation. MWHD: Microwave-assisted hydrodistillation. Different letters in the column indicate significant differences. A $p$ value < 0.05 indicates that the effect is significant with a 95% confidence level. F-value.

**Table S3.** Quantification of the compounds present in Java type citronella EOs, according to plant crop locations.

| Compound | Range, mg/kg | Linear equation | $R^2$ | LOD, mg/kg | LOQ, mg/kg | mg compound /kg EO, mean x $10^3$ ± SD ($n$ = 3) | | | | |
|---|---|---|---|---|---|---|---|---|---|---|
| | | | | | | Barbosa | Bucaramanga* | Chipatá | Puente Nacional | Vélez |
| Limonene | 260-2090 | y = 0.753x-19.778 | 0.9934 | 5 | 20 | 36 ± 2 | 20 ± 6 | 24 ± 1 | 40 ± 8 | 30 ± 2 |
| Linalool | 10-70 | y = 0.615x+0.858 | 0.9955 | 5 | 20 | 10 ± 1 | 6 ± 1 | 7.6 ± 0.4 | 13 ± 3 | 7.4 ± 0.2 |
| Citronellal | 2660-22560 | y = 0.478x+499.422 | 0.9940 | 5 | 20 | 550 ± 132 | 600 ± 48 | 340 ± 48 | 650 ± 167 | 320 ± 10 |
| Citronellol | 115-2090 | y = 0.730x-17.112 | 0.9955 | 5 | 20 | 160 ± 26 | 120 ± 15 | 160 ± 33 | 270 ± 21 | 130 ± 18 |
| Geraniol | 115-2090 | y = 0.778x-22.539 | 0.9955 | 5 | 20 | 300 ± 106 | 200 ± 46 | 210 ± 4 | 300 ± 97 | 191 ± 4 |
| Geranial [a] | 2660-22560 | y = 0.478x+499.422 | 0.9940 | 5 | 20 | 13 ± 1 | 10 ± 3 | 13 ± 1 | 11 ± 2 | 12.24 ± 0.04 |
| Citronellyl acetate [a] | 2660-22560 | y = 0.478x+499.422 | 0.9940 | 5 | 20 | 20 ± 4 | 20 ± 7 | 44 ± 2 | 24 ± 1 | 28 ± 1 |
| Eugenol [b] | 10-70 | y = 0.586x-0.225 | 0.9918 | 5 | 20 | 15 ± 1 | 15 ± 2 | 13 ± 2 | 16 ± 6 | 10 ± 1 |
| Geranyl acetate | 30-370 | y = 0.856x-7.703 | 0.9906 | 40 | 120 | 20 ± 7 | 17 ± 4 | 40 ± 5 | 23 ± 3 | 22 ± 3 |
| β-Elemene [c] | 10-70 | y = 0.717x+1.269 | 0.9931 | 10 | 20 | 6.0 ± 0.9 | 7 ± 3 | 5.5 ± 0.3 | 7 ± 2 | 4.0 ± 0.1 |
| Germacrene D [c] | 10-70 | y = 0.717x+1.269 | 0.9931 | 10 | 20 | 13 ± 1 | 14 ± 3 | 14 ± 2 | 20 ± 3 | 10.5 ± 0.3 |
| δ-Cadinene [c] | 10-70 | y = 0.717x+1.269 | 0.9931 | 10 | 20 | 5 ± 1 | 9 ± 3 | 8 ± 3 | 4 ± 1 | 6.0 ± 0.3 |
| Elemol [d] | 30-260 | y = 0.870x-7.665 | 0.9958 | 20 | 50 | 20 ± 4 | 30 ± 13 | 19 ± 4 | 20 ± 6 | 10 ± 1 |
| Germacrene D-4-ol [d] | 30-260 | y = 0.870x-7.665 | 0.9958 | 20 | 50 | 34 ± 10 | 40 ± 16 | 24 ± 7 | 35 ± 4 | 17.1 ± 0.2 |
| α-Cadinol [d] | 30-260 | y = 0.870x-7.665 | 0.9958 | 20 | 50 | 4.0 ± 0.8 | 11 ± 3 | 6 ± 2 | 3.4 ± 0.1 | 4.4 ± 0.4 |

[a] Quantification expressed as equivalents of citronellal (90%). [b] Quantification expressed as equivalents of geraniol (98%). [c] Quantification expressed as equivalents of (*E*)-β-caryophyllene (98.5%). [d] Quantification expressed as equivalents of caryophyllene oxide (95%). *$n$ = 5. LOD: Limit of detection. LOQ: Limit of quantification.

**Table S4.** Results of the analysis of variance used to evaluate the effect of plant material origin on the chemical composition of the EOs, distilled by MWHD, from Java type citronella plants.

| Compound | mg compound /kg EO | | | | | F | $p$ |
|---|---|---|---|---|---|---|---|
| | Barbosa | Bucaramanga | Chipatá | Puente Nacional | Vélez | | |
| Limonene | B,C | A | A,B | C | A,B,C | 7.18 | 0.0070 |
| Linalool | A,B | A | A | B | A | 8.89 | 0.0025 |
| Citronellal | A,B | A,B | A | B | A | 6.47 | 0.0063 |
| Citronellol | A | A | A | B | A | 9.66 | 0.0010 |
| Geraniol | A,B | A,B | A,B | B | A | 3.00 | 0.0626 |
| Geranial | A | A | A | A | A | 1.41 | 0.2952 |
| Citronellyl acetate | A | A | B | A | A | 10.06 | 0.0008 |
| Eugenol | A | A | A | A | A | 2.24 | 0.1305 |
| Geranyl acetate | A,B | A | B | A,B | A | 6.37 | 0.0055 |
| β-Elemene | A | A | A | A | A | 1.05 | 0.4339 |
| Germacrene D | A | A | A | B | A | 8.25 | 0.0019 |
| δ-Cadinene | A | A | A | A | A | 2.86 | 0.0751 |
| Elemol | A,B | B | A,B | A,B | A | 3.50 | 0.0448 |
| Germacrene D-4-ol | A | A | A | A | A | 2.61 | 0.0940 |
| α-Cadinol | A | B | A,B | A | A | 7.58 | 0.0028 |

Different letters in the column indicate significant differences. A $p$ value < 0.05 indicates that the effect is significant with a 95% confidence level. F-value.

**Table S5.** Quantification of the compounds present in palmarosa EOs, according to plant crop locations.

| Compound | Range, mg/kg | Linear equation | $R^2$ | LOD, mg/kg | LOQ, mg/kg | mg compound /kg EO, mean x $10^3$ ± SD ($n$ = 3) | | | | |
|---|---|---|---|---|---|---|---|---|---|---|
| | | | | | | Barbosa | Bucaramanga* | Chipatá | Puente Nacional | Vélez |
| β-Myrcene | 13-226 | y = 0.480x+0.554 | 0.9939 | 10 | 30 | 3.4 ± 0.7 | 3.0 ± 0.5 | 11 ± 5 | 6 ± 3 | 5.0 ± 0.3 |
| (*Z*)-β-Ocimene [a] | 13-226 | y = 0.480x+0.554 | 0.9939 | 10 | 30 | 4 ± 1 | 5 ± 1 | 5.0 ± 0.2 | 4 ± 1 | 5 ± 1 |
| (*E*)-β-Ocimene [a] | 13-226 | y = 0.480x+0.554 | 0.9939 | 10 | 30 | 20 ± 10 | 20 ± 5 | 25 ± 2 | 20 ± 2 | 31.0 ± 0.7 |
| Linalool | 115-2090 | y = 0.781x-14.411 | 0.9963 | 5 | 20 | 30 ± 9 | 19 ± 5 | 40 ± 6 | 34 ± 3 | 37 ± 2 |
| Nerol | 10-100 | y = 0.667x+0.806 | 0.9962 | 5 | 20 | 2.3 ± 0.3 | <LOQ | 4.5 ± 0.4 | 4 ± 1 | 4 ± 2 |
| Neral [b] | 10-70 | y = 0.500x+1.065 | 0.9930 | 5 | 20 | 3 ± 1 | 3.0 ± 0.7 | 4 ± 1 | 3.0 ± 0.9 | 3.0 ± 0.2 |
| Geraniol | 2932-22560 | y = 0.846x-666.874 | 0.9981 | 5 | 20 | 830 ± 38 | 900 ± 147 | 850 ± 38 | 940 ± 61 | 960 ± 126 |
| Geranial [b] | 10-70 | y = 0.500x+1.065 | 0.9930 | 5 | 20 | 9 ± 2 | 11 ± 5 | 11 ± 3 | 7 ± 1 | 11.0 ± 0.8 |
| Geranyl acetate | 30-370 | y = 0.856x-7.703 | 0.9906 | 40 | 120 | 60 ± 14 | 60 ± 11 | 40 ± 13 | 49 ± 1 | 70 ± 19 |
| (*E*)-β-Caryophyllene | 10-70 | y = 0.717x+1.269 | 0.9931 | 10 | 20 | 5 ± 1 | 6 ± 1 | 7 ± 1 | 4.0 ± 0.8 | 5.0 ± 0.7 |
| (2*E*,6*Z*-)-Farnesol | 10-260 | y = 0.696x-2.658 | 0.9934 | 20 | 70 | 5.0 ± 0.4 | 10 ± 7 | 6 ± 1 | 5 ± 1 | 5 ± 1 |

[a] Quantification expressed as equivalents of β-myrcene (94%). [b] Quantification expressed as equivalents of citronellal (90%). *$n$ = 5. LOD: Limit of detection. LOQ: Limit of quantification.

**Table S6.** Results of the analysis of variance used to evaluate the effect of plant material origin on the chemical composition of the EOs, distilled by MWHD, from palmarosa plants.

| Compound | mg compound /kg EO | | | | | F | *p* |
|---|---|---|---|---|---|---|---|
| | Barbosa | Bucaramanga | Chipatá | Puente Nacional | Vélez | | |
| β-Myrcene | A,B | A | D | C | B,C | 58.04 | <0.0001 |
| (*Z*)-β-Ocimene | A | A | A | A | A | 0.06 | 0.9915 |
| (*E*)-β-Ocimene | A | A | A | A | A | 1.01 | 0.4398 |
| Linalool | A,B | A | B | B | B | 9.10 | 0.0013 |
| Nerol | A | * | A | A | A | 1.52 | 0.2831 |
| Neral | A,B | A | B | A,B | A,B | 3.43 | 0.0432 |
| Geraniol | A | A | A | A | A | 1.43 | 0.2832 |
| Geranial | A | A | A | A | A | 0.51 | 0.7320 |
| Geranyl acetate | A | A | A | A | A | 0.54 | 0.7100 |
| (*E*)-β-Caryophyllene | A,B | A,B | B | A | A,B | 3.73 | 0.0338 |
| (2*E*,6*Z*-)-Farnesol | A | B | A | A | A | 7.18 | 0.0034 |

Different letters in the column indicate significant differences. A $p$ value < 0.05 indicates that the effect is significant with a 95% confidence level. F-value. ***Table S5** (<LOQ).

**Table S7.** Physicochemical properties of Java type citronella and palmarosa EOs, distilled in Santander, compared to the ISO 3848:2016 and ISO 4727:2021 data.

| Physicochemical properties | ISO 3848:2016 [16] Java type citronella | | ISO 4727:2021 [17] Palmarosa | | Java type citronella EO | Palmarosa EO |
|---|---|---|---|---|---|---|
| | Min. | Max. | Min. | Max. | Value ± SD ($n$ = 16) | |
| Refractive index [a] | 1.466 | 1.477 | 1.471 | 1.478 | 1.466 ± 0.004 | 1.472 ± 0.002 |
| Relative density [b], at 20 °C | 0.880 | 0.902 | 0.880 | 0.894 | 0.89 ± 0.01 | 0.888 ± 0.002 |
| Optical rotation [c], degrees | -5.0 | +1 | -1 | +3 | -2 ± 1 | +1.5 ± 0.3 |
| Acid value [d], mg KOH/g EO | N.R. | | 0 | 1.0 | 1.4 ± 0.6 | 1.2 ± 0.6 |
| Ester value [e], mg KOH/g EO | N.R. | | 7 | 36 | 34 ± 8 | 35 ± 10 |
| Freezing point [f], °C | N.R. | | N.R. | | -69 ± 3 | -65 ± 3 |
| Flash point [g], °C | 81 | | N.R. | | 83 ± 4 | 98 ± 7 |

| Water content [h], % | N.R. | N.R. | 1.5 ± 0.3 | 3.4 ± 0.5 |
|---|---|---|---|---|

| Data | ISO 3848:2016 [16] | ISO 4727:2021 [17] | Miscibility in ethanol [i], % v/v | | | | | |
|---|---|---|---|---|---|---|---|---|
| | | | Java type citronella EO | | | Palmarosa EO | | |
| | *C. winterianus* | *C. martinii* | 70% | 80% | 90% | 60% | 70% | 80% |
| Turbidity, mL | Two parts of ethanol (80%) for one part of essential oil. | Two parts of ethanol (70%) for one part of essential oil. | 0.1 | 0.1 | - | 0.2 | 0.1 | 0.1 |
| Turbidity disappears, mL | | | 0.5 | 0.3 | - | 0.7 | 0.3 | 0.2 |
| Opalescence, mL | | | 0.5-12.8 | 0.3-5.1 | - | 0.7-3.0 | 0.3-1.4 | 0.2-1.3 |
| Cloudiness, mL | | | 12.8-20 | 5.1-6.4 | 1.1-1.2 | 3.0-4.1 | 1.4-1.8 | - |
| Miscibility, mL | | | - | 6.4-20 | 1.2-20 | 4.1-20 | 1.8-20 | 1.3-20 |

[a] ISO 280:1998 [75]. [b] ISO 279:1998 [69]. [c] ISO 592:1998 [71]. [d] ISO 1242:1999 [76]. [e] ISO 709:2001 [77]. [f] ISO 1041:1973 [74]. [g] ISO/TR 11018:1997 [72]. [h] ISO 11021:1999 [73]. [i] ISO 875:1999 [70]. N.R. Value not reported in ISO 3848:2016 [16] or ISO ISO 4727:2021 [17].

**Table S8**. Chemical composition of the EOs, distilled by S.D., from Java type citronella and palmarosa plants harvested in different municipalities of Santander (Colombia).

| Compound | GC/FID relative peak area, % mean (± SD, *n* = 16) | | | |
|---|---|---|---|---|
| | ISO 3848:2016 [16] Java type citronella | ISO 4727:2021 [17] Palmarosa | Java type citronella EO | Palmarosa EO |
| β-Myrcene | - | 0.1 - 0.5 | - | 0.4 - 0.2 |
| Limonene | 2 -5 | 0.1 - 1.0 | 3.0 ± 0.5 | 0.12 ± 0.06 |
| (*E*)-β-Ocimene | - | 0.2 - 2.0 | - | 1.5 ± 0.3 |
| Linalool | 0.5 - 1.5 | 1.5 - 4.0 | 0.8 ± 0.1 | 3.1 ± 0.7 |
| Nerol | - | 0.2 - 1.0 | - | 0.3 ± 0.2 |
| Citronellal | 31 - 40 | - | 33 ± 7 | - |
| Citronellol | 8.5 - 14 | - | 17 ± 3 | - |
| Neral | - | 0.05 - 0.03 | 0.4 ± 0.1 | 0.2 ± 0.1 |
| Geraniol | 20 - 25 | 77 - 85 | 27 ± 5 | 85 ± 2 |
| Geranial | 0.3 - 1.0 | 0.1 - 0.6 | 0.6 ± 0.2 | 0.6 ± 0.4 |
| Citronellyl acetate | 2.0 - 4.0 | - | 2.0 ± 0.9 | - |
| Eugenol | 0.5 - 1.0 | - | 1.2 ± 0.4 | - |
| Geranyl acetate | 2.5 - 5.5 | 5 - 13 | 2 ± 1 | 6 ± 2 |
| β-Elemene | 0.7 - 2.5 | - | 0.9 ± 0.8 | - |
| Germacrene D | 1.5 - 3.0 | - | 1.2 ± 0.3 | - |
| Elemol | 1.3 - 4.8 | - | 4 ± 1 | - |
| Germacrene D 4-ol | - | - | 4 ± 1 | - |
| (*E*)-β-Caryophyllene | - | 1.0 - 2.5 | - | 0.4 ± 0.1 |
| (2*E*,6*Z*)-Farnesol | - | *tr.* - 1.5 | - | 0.5 ± 0.2 |
| Geranyl hexanoate | - | 0.2 - 1.0 | - | 0.3 ± 0.1 |

**Table S9**. Yields of hydroalcoholic extracts of Java type citronella and palmarosa plants, cultivated in Santander (Colombia).

| Species | Vegetal material | Harvesting place | Phenological stage | Plant material treatment | Yield, % ± SD (*n* = 3) |
|---|---|---|---|---|---|
| Citronella | Before distillation | Bucaramanga, Santander | Vegetative | Dried | 11 ± 1 |
| | Postdistillation waste | | | | 11 ± 3 |
| Palmarosa | Before distillation | Bucaramanga, Santander | Post-flowering | Dried | 6 ± 1 |
| | Postdistillation waste | | | | 4 ± 1 |

**Table S10.** Results of the analysis of variance used to evaluate the effect of plant material, before distillation or postdistillation waste, on yields of hydroalcoholic extracts of Java type citronella and palmarosa.

| **Yields** | | | | | | | |
|---|---|---|---|---|---|---|---|
| **Java type citronella** | | | | **Palmarosa** | | | |
| **Before distillation** | **Residual biomass** | **F** | ***p*** | **Before distillation** | **Residual biomass** | **F** | ***p*** |
| A | A | 0.02 | 0.9090 | A | A | 2.3 | 0.2264 |

Different letters in the column indicate significant differences. A *p* value < 0.05 indicates that the effect is significant with a 95% confidence level. F-value.

**Table S11.** Exact masses of protonated $[M + H]^+$ and deprotonated $[M – H]^-$ molecules, identified by UHPLC-ESI-Orbitrap-MS, in the *C. winterianus* and *C. martinii* hydroalcoholic extracts.

| Peak N° Fig. 2 | Compound | Formula | Exp. masses, *m/z* (I, %) | Δppm | HCD, eV | Product-ions | Formula | *m/z* (I, %) | Ref. |
|---|---|---|---|---|---|---|---|---|---|
| 1 | Caffeoyl quinic acid isomer [a] | $C_{16}H_{18}O_9$ | $[M – H]^-$ 353.08792 (100) | 1.21 | 10 | $[(M – H) – C_9H_6O_3]^-$ | $C_7H_{11}O_6$ | 191.05501 (86) | - |
| | | | | | | $[(M – H) – C_7H_{10}O_5]^-$ | $C_9H_7O_4$ | 179.03430 (53) | |
| | | | | | | $[(M – H) – C_8H_{10}O_7]^-$ | $C_8H_7O_2$ | 135.04430 (10) | |
| 2 | Hydroxy benzoic acid isomer [a] | $C_7H_6O_3$ | $[M – H]^-$ 137.02342 (2) | 0.01 | 20 | $[(M – H) – CO_2]^-$ | $C_6H_5O$ | 93.03350 (100) | - |
| 3 | *p*-Hydroxy benzoic acid [a,b,c] | $C_7H_6O_3$ | $[M – H]^-$ 137.02353 (46) | 0.20 | 10 | $[(M – H) – CO_2]^-$ | $C_6H_5O$ | 93.03351 (100) | [27] |
| 4 | Ferulyol quinic acid isomer [a] | $C_{17}H_{20}O_9$ | $[M – H]^-$ 367.10315 (9) | 0.76 | 20 | $[(M – H) – C_7H_{10}O_5]^-$ | $C_{10}H_9O_4$ | 193.04993 (100) | - |
| | | | | | | $[(M – H) – C_{10}H_8O_3]^-$ | $C_7H_{11}O_6$ | 191.05539 (4) | |
| | | | | | | $[(M – H) – C_{10}H_{10}O_4]^-$ | $C_7H_9O_5$ | 173.04468 (5) | |
| 5 | 3-Caffeoyl quinic acid [a,b,c] | $C_{16}H_{18}O_9$ | $[M – H]^-$ 353.08792 (14) | 1.21 | 10 | $[(M – H) – C_9H_6O_3]^-$ | $C_7H_{11}O_6$ | 191.05501 (100) | [27] |
| 6 | Coumaric acid isomer [a] | $C_9H_8O_3$ | $[M – H]^-$ 163.03905 (7) | 0.07 | 20 | $[(M – H) – CO_2]^-$ | $C_8H_7O$ | 119.04916 (100) | - |
| 7 | 4-Caffeoyl quinic acid [a,c] | $C_{16}H_{18}O_9$ | $[M – H]^-$ 353.08798 (85) | 1.18 | 10 | $[(M – H) – C_9H_6O_3]^-$ | $C_7H_{11}O_6$ | 191.05501 (49) | - |
| | | | | | | $[(M – H) – C_7H_{10}O_5]^-$ | $C_9H_7O_4$ | 179.03430 (73) | |
| | | | | | | $[(M – H) – C_9H_8O_4]^-$ | $C_7H_9O_5$ | 173.04483 (100) | |
| | | | | | | $[(M – H) – C_8H_{10}O_7]^-$ | $C_8H_7O_2$ | 135.04430 (8) | |
| 8 | Caffeic acid [a,b,c] | $C_9H_8O_4$ | $[M – H]^-$ 179.03396 (34) | 0.39 | 10 | $[(M – H) – CO_2]^-$ | $C_8H_7O_2$ | 135.04396 (100) | [27] |
| 9 | Caffeoyl quinic acid isomer [a,b] | $C_{16}H_{18}O_9$ | $[M – H]^-$ 353.08820 (21) | 1.48 | 10 | $[(M – H) – C_9H_6O_3]^-$ | $C_7H_{11}O_6$ | 191.05545 (100) | [58] |
| | | | | | | $[(M – H) – C_7H_{10}O_5]^-$ | $C_9H_7O_4$ | 179.03430 (3) | |
| | | | | | | $[(M – H) – C_9H_8O_4]^-$ | $C_7H_9O_5$ | 173.04483 (3) | |
| 10 | Luteolin-*C*-hexoside-*C*-pentoside [a] | $C_{26}H_{28}O_{15}$ | $[M + H]^+$ 581.14978 (12) | 0.91 | 20 | $[(M + H) – H_2O]^+$ | $C_{26}H_{27}O_{14}$ | 563.1394 (53) | - |
| | | | | | | $[(M + H) – 2H_2O]^+$ | $C_{26}H_{25}O_{13}$ | 545.12891 (83) | |
| | | | | | | $[(M + H) – 3H_2O]^+$ | $C_{26}H_{23}O_{12}$ | 527.11804 (100) | |
| | | | | | | $[(M + H) – C_4H_8O_4]^+$ | $C_{22}H_{21}O_{11}$ | 461.10925 (49) | |

**Table S11**. Continued.

| | | | | | | | | | |
|---|---|---|---|---|---|---|---|---|---|
| | | | | | | $[(M + H) – H_2O – C_4H_8O_4]^+$ | $C_{22}H_{19}O_{10}$ | 443.09717 (80) | |
| | | | | | | $[(M + H) – 2H_2O – C_4H_8O_4]^+$ | $C_{22}H_{17}O_9$ | 425.08646 (69) | |
| | | | | | | $[(M + H) – 2H_2O – C_5H_8O_4]^+$ | $C_{21}H_{17}O_9$ | 413.08627 (84) | |
| | | | | | | $[(M + H) – 2H_2O – C_5H_{10}O_5]^+$ | $C_{21}H_{15}O_8$ | 395.07590 (52) | |
| | | | | | | $[(M + H) – 2C_4H_8O_4]^+$ | $C_{18}H_{13}O_7$ | 341.06531 (19) | |
| | | | | | | $[(M + H) – C_4H_8O_4 – C_5H_{10}O_5]^+$ | $C_{17}H_{11}O_6$ | 311.05508 (1) | |
| 11 | Coumaric acid isomer [a] | $C_9H_8O_3$ | $[M – H]^-$ 163.03917 (100) | 0.19 | 10 | $[(M – H) – CO]^-$ | $C_8H_7O_2$ | 135.04410 (8) | - |
| | | | | | | $[(M – H) – CO_2]^-$ | $C_8H_7O$ | 119.04935 (3) | |
| 12 | Ferulyol quinic acid isomer [a] | $C_{17}H_{20}O_9$ | $[M – H]^-$ 367.10318 (6) | 1.03 | 20 | $[(M – H) – C_7H_{10}O_5]^-$ | $C_{10}H_9O_4$ | 193.04990 (9) | - |
| | | | | | | $[(M – H) – C_{10}H_8O_3]^-$ | $C_7H_{11}O_6$ | 191.05539 (100) | |
| | | | | | | $[(M – H) – C_{10}H_{10}O_4]^-$ | $C_7H_9O_5$ | 173.04469 (38) | |
| 13 | Apigenin-*C*,*C*-dihexoside [a] | $C_{27}H_{30}O_{15}$ | $[M + H]^+$ 595.16498 (13) | 0.77 | 20 | $[(M + H) – H_2O]^+$ | $C_{27}H_{29}O_{14}$ | 577.15479 (26) | - |
| | | | | | | $[(M + H) – 2H_2O]^+$ | $C_{27}H_{27}O_{13}$ | 559.14429 (49) | |
| | | | | | | $[(M + H) – 3H_2O]^+$ | $C_{27}H_{25}O_{12}$ | 541.13379 (69) | |
| | | | | | | $[(M + H) – 2H_2O – CH_2O]^+$ | $C_{26}H_{25}O_{12}$ | 529.13385 (29) | |
| | | | | | | $[(M + H) – 3H_2O – CH_2O]^+$ | $C_{26}H_{23}O_{11}$ | 511.12311 (27) | |
| | | | | | | $[(M + H) – H_2O – C_4H_8O_4]^+$ | $C_{23}H_{21}O_{10}$ | 457.11246 (100) | |
| | | | | | | $[(M + H) – 2H_2O – C_4H_8O_4]^+$ | $C_{23}H_{19}O_9$ | 439.10181 (69) | |
| | | | | | | $[(M + H) – 2H_2O – C_4H_8O_4 – CH_2O]^+$ | $C_{22}H_{17}O_8$ | 409.09128 (21) | |
| 14 | Luteolin-*C*-hexoside-*C*-pentoside isomer [a,b] | $C_{26}H_{28}O_{15}$ | $[M + H]^+$ 581.14978 (23) | 0.32 | 20 | $[(M + H) – H_2O]^+$ | $C_{26}H_{27}O_{14}$ | 563.13892 (80) | [58] |
| | | | | | | $[(M + H) – 2H_2O]^+$ | $C_{26}H_{25}O_{13}$ | 545.12836 (100) | |
| | | | | | | $[(M + H) – 3H_2O]^+$ | $C_{26}H_{23}O_{12}$ | 527.11786 (99) | |
| | | | | | | $[(M + H) – C_4H_8O_4]^+$ | $C_{22}H_{21}O_{11}$ | 461.10812 (48) | |
| | | | | | | $[(M + H) – H_2O – C_4H_8O_4]^+$ | $C_{22}H_{19}O_{10}$ | 443.09663 (96) | |
| | | | | | | $[(M + H) – 2H_2O – C_4H_8O_4]^+$ | $C_{22}H_{17}O_9$ | 425.08609 (68) | |
| | | | | | | $[(M + H) – 2H_2O – C_5H_8O_4]^+$ | $C_{21}H_{17}O_9$ | 413.08609 (47) | |
| | | | | | | $[(M + H) – 2H_2O – C_5H_{10}O_5]^+$ | $C_{21}H_{15}O_8$ | 395.07559 (38) | |
| | | | | | | $[(M + H) – C_4H_8O_4 – C_4H_8O_4]^+$ | $C_{18}H_{13}O_7$ | 341.06503 (13) | |
| | | | | | | $[(M + H) – C_4H_8O_4 – C_5H_{10}O_5]^+$ | $C_{17}H_{11}O_6$ | 311.05386 (1) | |

**Table S11**. Continued.

| | | | | | | | | | |
|---|---|---|---|---|---|---|---|---|---|
| 15 | Luteolin-*C*,*C*-dipentoside isomer | $C_{25}H_{26}O_{14}$ | | 0.05 | 30 | $[(M + H) – H_2O]^+$ | $C_{25}H_{25}O_{13}$ | 533.12854 (1) | |

| | | | | | | | | | |
|---|---|---|---|---|---|---|---|---|---|
| | | | [M + H]+ 551.13959 (1) | | | $[(M + H) - 2H_2O]^+$ | $C_{25}H_{23}O_{12}$ | 515.11792 (5) | - |
| | | | | | | $[(M + H) - 3H_2O]^+$ | $C_{25}H_{21}O_{11}$ | 497.10706 (34) | |
| | | | | | | $[(M + H) - 3H_2O - CH_2O]^+$ | $C_{24}H_{19}O_{10}$ | 467.08633 (66) | |
| | | | | | | $[(M + H) - H_2O - C_3H_6O_3]^+$ | $C_{22}H_{19}O_{10}$ | 443.09839 (21) | |
| | | | | | | $[(M + H) - 2H_2O - C_3H_6O_3]^+$ | $C_{22}H_{17}O_9$ | 425.08615 (51) | |
| | | | | | | $[(M + H) - H_2O - C_4H_8O_4]^+$ | $C_{21}H_{17}O_9$ | 413.08615 (18) | |
| | | | | | | $[(M + H) - 2H_2O - C_4H_8O_4]^+$ | $C_{21}H_{15}O_8$ | 395.07568 (100) | |
| | | | | | | $[(M + H) - 3H_2O - C_5H_8O_4]^+$ | $C_{20}H_{13}O_7$ | 365.06470 (45) | |
| | | | | | | $[(M + H) - C_4H_8O_4 - C_4H_8O_4]^+$ | $C_{17}H_{11}O_6$ | 311.05469 (20) | |
| | | | | | | $[(M + H) - C_4H_8O_4 - C_5H_8O_4]^+$ | $C_{16}H_{11}O_6$ | 299.05460 (1) | |
| 16 | Ferulyol quinic acid isomer [a] | $C_{17}H_{20}O_9$ | $[M - H]^-$ 367.10303 (7) | 0.88 | 20 | $[(M - H) - C_7H_{10}O_5]^-$ | $C_{10}H_9O_4$ | 193.05003 (10) | - |
| | | | | | | $[(M - H) - C_{10}H_8O_3]^-$ | $C_7H_{11}O_6$ | 191.05537 (100) | |
| | | | | | | $[(M - H) - C_{10}H_{10}O_4]^-$ | $C_7H_9O_5$ | 173.04474 (34) | |
| 17 | *p*-Coumaric acid [a,b,c] | $C_9H_8O_3$ | $[M - H]^-$ 163.03915 (11) | 0.18 | 10 | $[(M - H) - CO_2]^-$ | $C_8H_7O$ | 119.04892 (100) | [27] |
| 18 | Apigenin-*C*-hexoside-*C*-pentoside isomer | $C_{26}H_{28}O_{14}$ | $[M + H]^+$ 565.15479 (1) | 0.70 | 30 | $[(M + H) - H_2O]^+$ | $C_{26}H_{27}O_{13}$ | 547.14178 (1) | - |
| | | | | | | $[(M + H) - 2H_2O]^+$ | $C_{26}H_{25}O_{12}$ | 529.13397 (6) | |
| | | | | | | $[(M + H) - 3H_2O]^+$ | $C_{26}H_{23}O_{11}$ | 511.12283 (18) | |
| | | | | | | $[(M + H) - C_4H_8O_4]^+$ | $C_{22}H_{21}O_{10}$ | 445.08957 (18) | |
| | | | | | | $[(M + H) - 2H_2O - C_4H_8O_4]^+$ | $C_{22}H_{17}O_8$ | 409.09106 (55) | |
| | | | | | | $[(M + H) - 2H_2O - C_5H_{10}O_5]^+$ | $C_{21}H_{15}O_7$ | 379.08054 (100) | |
| | | | | | | $[(M + H) - 2H_2O - C_4H_8O_4 - C_2H_4O_2]^+$ | $C_{20}H_{13}O_6$ | 349.07004 (38) | |
| | | | | | | $[(M + H) - 2C_4H_8O_4]^+$ | $C_{18}H_{13}O_6$ | 325.07004 (48) | |
| | | | | | | $[(M + H) - C_4H_8O_4 - C_5H_{10}O_5]^+$ | $C_{17}H_{11}O_5$ | 295.05954 (25) | |
| | | | | | | $[(M + H) - C_5H_8O_4 - C_5H_{10}O_5]^+$ | $C_{16}H_{11}O_5$ | 283.05954 (2) | |
| 19 | Luteolin-6-*C*-glucoside [a] | $C_{21}H_{20}O_{11}$ | $[M + H]^+$ 449.10696 (40) | 0.87 | 20 | $[(M + H) - H_2O]^+$ | $C_{21}H_{19}O_{10}$ | 431.09644 (60) | - |
| | | | | | | $[(M + H) - 2H_2O]^+$ | $C_{21}H_{17}O_9$ | 413.08588 (71) | |
| | | | | | | $[(M + H) - 3H_2O]^+$ | $C_{21}H_{15}O_8$ | 395.07541 (51) | |
| | | | | | | $[(M + H) - 2H_2O - CH_2O]^+$ | $C_{20}H_{15}O_8$ | 383.07538 (77) | |
| | | | | | | $[(M + H) - 2H_2O - C_2H_4O_2]^+$ | $C_{19}H_{13}O_7$ | 353.06485 (88) | |

**Table S11**. Continued.

| | | | | | | | | | |
|---|---|---|---|---|---|---|---|---|---|
| | | | | | | $[(M + H) - C_4H_8O_4]^+$ | $C_{17}H_{13}O_7$ | 329.06558 (100) | |
| | | | | | | $[(M + H) - C_5H_{10}O_5]^+$ | $C_{16}H_{11}O_6$ | 299.05438 (68) | |

| | | | | | | | | | |
|---|---|---|---|---|---|---|---|---|---|
| | | | | | | $[(M + H) – C_6H_{10}O_5]^+$ | $C_{15}H_{11}O_6$ | 287.05438 (5) | |
| 20 | Ferulic acid [a,b,c] | $C_{10}H_{10}O_4$ | $[M – H]^–$ 193.04997 (51) | 0.44 | 10 | $[(M – H) – CH_3]^{–•}$ | $C_9H_6O_4$ | 178.02647 (100) | [27] |
| | | | | | | $[(M – H) – CO_2]^–$ | $C_9H_9O_2$ | 149.05991 (35) | |
| | | | | | | $[(M – H) – C_3H_2O]^–$ | $C_7H_7O_3$ | 139.03911 (6) | |
| | | | | | | $[(M – H) – C_3H_4O]^–$ | $C_7H_5O_3$ | 137.02347 (16) | |
| | | | | | | $[(M – H) – C_2H_3O_2]^{–•}$ | $C_8H_6O_2$ | 134.03639 (87) | |
| 21 | N.I. (**Fig. S1**) | $C_{16}H_{18}O_9$ | $[M – H]^–$ 353.08777 (2) | 3.00 | 10 | $[(M – H) – H_2O]^–$ | $C_{16}H_{15}O_8$ | 335.07718 (1) | - |
| | | | | | | $[(M – H) – C_6H_8O_5]^–$ | $C_{10}H_9O_4$ | 193.05006 (5) | |
| | | | | | | $[(M – H) – C_{10}H_{10}O_4]^–$ | $C_6H_7O_5$ | 159.02910 (100) | |
| 22 | Luteolin-*O*-desoxyhexosyl-*C*-hexoside [a] | $C_{27}H_{30}O_{15}$ | $[M + H]^+$ 595.16473 (8) | 1.01 | 20 | $[(M + H) – H_2O]^+$ | $C_{27}H_{29}O_{14}$ | 577.15503 (11) | - |
| | | | | | | $[(M + H) – 2H_2O]^+$ | $C_{27}H_{27}O_{13}$ | 559.14398 (15) | |
| | | | | | | $[(M + H) – C_6H_{10}O_4]^+$ | $C_{21}H_{21}O_{11}$ | 449.10730 (100) | |
| | | | | | | $[(M + H) – H_2O – C_6H_{10}O_4]^+$ | $C_{21}H_{19}O_{10}$ | 431.09671 (67) | |
| | | | | | | $[(M + H) – 2H_2O – C_6H_{10}O_4]^+$ | $C_{21}H_{17}O_9$ | 413.10184 (35) | |
| | | | | | | $[(M + H) – C_6H_{10}O_4 – C_4H_8O_4]^+$ | $C_{17}H_{13}O_7$ | 329.06512 (47) | |
| | | | | | | $[(M + H) – C_6H_{10}O_4 – C_5H_{10}O_5]^+$ | $C_{16}H_{11}O_6$ | 299.05453 (12) | |
| 23 | Luteolin-*C,C*-dipentoside isomer | $C_{25}H_{26}O_{14}$ | $[M + H]^+$ 551.13959 (2) | 2.50 | 30 | $[(M + H) – H_2O]^+$ | $C_{25}H_{25}O_{13}$ | 533.12634 (1) | - |
| | | | | | | $[(M + H) – 2H_2O]^+$ | $C_{25}H_{23}O_{12}$ | 515.11786 (5) | |
| | | | | | | $[(M + H) – 3H_2O]^+$ | $C_{25}H_{21}O_{11}$ | 497.10730 (53) | |
| | | | | | | $[(M + H) – 3H_2O – CH_2O]^+$ | $C_{24}H_{19}O_{10}$ | 467.08612 (80) | |
| | | | | | | $[(M + H) – 2H_2O – C_3H_6O_3]^+$ | $C_{22}H_{17}O_9$ | 425.08615 (69) | |
| | | | | | | $[(M + H) – H_2O – C_4H_8O_4]^+$ | $C_{21}H_{17}O_9$ | 413.08609 (26) | |
| | | | | | | $[(M + H) – 2H_2O – C_4H_8O_4]^+$ | $C_{21}H_{15}O_8$ | 395.07562 (100) | |
| | | | | | | $[(M + H) – 3H_2O – C_5H_8O_4]^+$ | $C_{20}H_{13}O_7$ | 365.06506 (43) | |
| | | | | | | $[(M + H) – C_4H_8O_4 – C_4H_8O_4]^+$ | $C_{17}H_{11}O_6$ | 311.05447 (19) | |
| | | | | | | $[(M + H) – C_4H_8O_4 – C_5H_8O_4]^+$ | $C_{16}H_{11}O_6$ | 299.05423 (1) | |
| 24 | Apigenin-8-*C*-glucoside [a,b,c] | $C_{21}H_{20}O_{10}$ | $[M + H]^+$ 433.11255 (100) | 0.40 | 20 | $[(M + H) – H_2O]^+$ | $C_{21}H_{19}O_9$ | 415.10211 (36) | [57] |
| | | | | | | $[(M + H) – 2H_2O]^+$ | $C_{21}H_{17}O_8$ | 397.09137 (20) | |

**Table S11**. Continued.

| | | | | | | | | | |
|---|---|---|---|---|---|---|---|---|---|
| | | | | | | $[(M + H) – 3H_2O]^+$ | $C_{21}H_{15}O_7$ | 379.08115 (4) | |
| | | | | | | $[(M + H) – 2H_2O – CH_2O]^+$ | $C_{20}H_{15}O_7$ | 367.08093 (8) | |
| | | | | | | $[(M + H) – 2H_2O – C_2H_4O_2]^+$ | $C_{19}H_{13}O_6$ | 337.07031 (4) | |
| | | | | | | $[(M + H) – C_4H_8O_4]^+$ | $C_{17}H_{13}O_6$ | 313.07047 (18) | |

| | | | | | | | | | |
|---|---|---|---|---|---|---|---|---|---|
| | | | | | | $[(M + H) - C_5H_{10}O_5]^+$ | $C_{16}H_{11}O_5$ | 283.05984 (3) | |
| | | | | | | $[(M + H) - C_6H_{10}O_5]^+$ | $C_{15}H_{11}O_5$ | 271.05981 (1) | |
| 25 | *o*-Hydroxy benzoic acid [a,b,c] | $C_7H_6O_3$ | $[M - H]^-$ 137.02345 (38) | 0.13 | 10 | $[(M - H) - CO_2]^-$ | $C_6H_5O$ | 93.03352 (100) | - |
| 26 | Apigenin-*C*-hexoside isomer[a] | $C_{21}H_{20}O_{10}$ | $[M + H]^+$ 433.11255 (16) | 1.99 | 20 | $[(M + H) - H_2O]^+$ | $C_{21}H_{19}O_9$ | 415.10172 (57) | - |
| | | | | | | $[(M + H) - 2H_2O]^+$ | $C_{21}H_{17}O_8$ | 397.09109 (69) | |
| | | | | | | $[(M + H) - 3H_2O]^+$ | $C_{21}H_{15}O_7$ | 379.08060 (43) | |
| | | | | | | $[(M + H) - 2H_2O - CH_2O]^+$ | $C_{20}H_{15}O_7$ | 367.08069 (61) | |
| | | | | | | $[(M + H) - 2H_2O - C_2H_4O_2]^+$ | $C_{19}H_{13}O_6$ | 337.07004 (77) | |
| | | | | | | $[(M + H) - C_4H_8O_4]^+$ | $C_{17}H_{13}O_6$ | 313.07013 (100) | |
| | | | | | | $[(M + H) - C_5H_{10}O_5]^+$ | $C_{16}H_{11}O_5$ | 283.05960 (49) | |
| 27 | Luteolin-*O*-rutinoside [a] | $C_{27}H_{30}O_{15}$ | $[M + H]^+$ 595.16522 (2) | 0.52 | 20 | $[(M + H) - C_6H_{10}O_4]^+$ | $C_{21}H_{21}O_{11}$ | 449.10727 (5) | - |
| | | | | | | $[(M + H) - H_2O - C_6H_{10}O_4]^+$ | $C_{21}H_{19}O_{10}$ | 431.09677 (3) | |
| | | | | | | $[(M + H) - C_6H_{10}O_4 - C_6H_{10}O_5]^+$ | $C_{15}H_{11}O_6$ | 287.05460 (100) | |
| 28 | Luteolin-*O*-hexoside-*C*-pentoside isomer | $C_{26}H_{28}O_{14}$ | $[M + H]^+$ 565.15491 (2) | 2.40 | 30 | $[(M + H) - C_6H_{10}O_4]^+$ | $C_{20}H_{19}O_{10}$ | 419.09665 (10) | - |
| | | | | | | $[(M + H) - C_6H_{10}O_4 - H_2O]^+$ | $C_{20}H_{17}O_9$ | 401.08627 (10) | |
| | | | | | | $[(M + H) - C_6H_{10}O_4 - 2H_2O]^+$ | $C_{20}H_{15}O_8$ | 383.07559 (100) | |
| | | | | | | $[(M + H) - C_6H_{10}O_4 - 2H_2O - CH_2O]^+$ | $C_{19}H_{13}O_7$ | 353.06500 (60) | |
| | | | | | | $[(M + H) - C_6H_{10}O_4 - C_4H_8O_4]^+$ | $C_{16}H_{11}O_6$ | 299.05344 (35) | |
| 29 | Luteolin [a,b,c] | $C_{15}H_{10}O_6$ | $[M + H]^+$ 287.05466 (100) | 0.32 | 50 | $[(M + H) - H_2O]^+$ | $C_{15}H_9O_5$ | 269.04385 (2) | [56] |
| | | | | | | $[(M + H) - C_8H_4O]^+$ | $C_7H_7O_5$ | 171.02878 (4) | |
| | | | | | | $[(M + H) - C_8H_6O_2]^+$ | $C_7H_5O_4$ | 153.01820 (10) | |
| | | | | | | $[(M + H) - C_7H_4O_4]^+$ | $C_8H_7O_2$ | 135.04393 (5) | |

**Table S11**. Continued.

| | | | | | | | | | |
|---|---|---|---|---|---|---|---|---|---|
| 30 | Tricin [a] | $C_{17}H_{14}O_7$ | $[M + H]^+$ 331.08081 (17) | 1.26 | 40 | $[(M + H) - CH_3]^{+\bullet}$ | $C_{16}H_{12}O_7$ | 316.05743 (4) | - |
| | | | | | | $[(M + H) - 2CH_3]^+$ | $C_{15}H_9O_7$ | 301.03384 (10) | |
| | | | | | | $[(M + H) - C_2H_4O]^+$ | $C_{15}H_{11}O_6$ | 287.05450 (11) | |
| | | | | | | $[(M + H) - C_2H_5O_2]^{+\bullet}$ | $C_{15}H_{10}O_5$ | 270.05194 (22) | |
| | | | | | | $[(M + H) - C_3H_5O_2]^{+\bullet}$ | $C_{14}H_{10}O_5$ | 258.05197 (4) | |

| | | |
|---|---|---|
| $[(M + H) – C_3H_5O_3]^{+\bullet}$ | $C_{14}H_{10}O_4$ | 242.05711 (4) |
| $[(M + H) – C_8H_9O_3]^{+\bullet}$ | $C_9H_6O_4$ | 178.02602 (1) |
| $[(M + H) – C_{10}H_{10}O_3]^{+}$ | $C_7H_5O_4$ | 153.05408 (1) |

[a] Identification based on the study of fragmentation pattern and their comparison with mass spectra from *HMDB* 4.0 and PCIDB databases. [b] Identification based on scientific literature data on species of genus *Cymbopogon* [27, 56-58].[c] Confirmatory identification based on the comparison of sample spectra with mass spectra and retention times ($t_R$) of standard substances of *p*-hydroxy benzoic (≥99%), *o*-hydroxy benzoic (99.98%), *p*-coumaric (≥98%), ferulic (≥99%), caffeic (≥98%), 3-caffeoyl quinic (≥99%), and 4-caffeoyl quinic (≥98%) acids, and apigenin-8-*C*-glucoside (≥95%) and luteolin (98%).

**Table S12.** Quantification of phenolic compounds, by UHPL-ESI-Orbitrap-MS, operated in SIM mode, present in the *Cymbopogon* spp. extracts studied.

| N° | Compound | mg compound/g extract ± SD (*n* = 3) | | | |
|---|---|---|---|---|---|
| | | Java type citronella | | Palmarosa | |
| | | Before distillation | Residual biomass | Before distillation | Residual biomass |
| 1 | Caffeoyl quinic acid isomer [a] | 1.65 ± 0.01 | 1.1 ± 0.7 | < LOD | 0.129 ± 0.002 |
| 2 | Hydroxy benzoic acid isomer [b] | < LOD | 1.0 ± 0.3 | 0.301 ± 0.008 | 0.9 ± 0.8 |
| 3 | *p*-Hydroxy benzoic acid | - | 0.2 ± 0.1 | 0.181 ± 0.009 | 2 ± 1 |
| 4 | Ferulyol quinic acid isomer [c] | 12 ± 1 | 0.7 ± 0.4 | 0.52 ± 0.02 | 0.103 ± 0.001 |
| 5 | 3-Caffeoyl quinic acid | 2.7 ± 0.1 | 2 ± 1 | 0.249 ± 0.004 | 0.15 ± 0.01 |
| 6 | Coumaric acid isomer [d] | < LOD | - | 0.067 ± 0.002 | - |
| 7 | 4-Caffeoyl quinic acid | 1.08 ± 0.03 | 3 ± 1 | - | 0.336 ± 0.006 |
| 8 | Caffeic acid | < LOD | < LOQ | 0.187 ± 0.006 | < LOD |
| 9 | Caffeoyl quinic acid isomer [a] | 0.288 ± 0.009 | 0.7 ± 0.5 | 0.249 ± 0.004 | - |
| 10 | Luteolin-*C*-hexoside-*C*-pentoside [e] | 0.309 ± 0.006 | 0.7 ± 0.5 | - | 0.400 ± 0.005 |
| 11 | Coumaric acid isomer [d] | 0.063 ± 0.003 | - | 1.13 ± 0.03 | - |
| 12 | Ferulyol quinic acid isomer [c] | 2.7 ± 0.2 | 1.4 ± 0.6 | 0.178 ± 0.005 | 0.095 ± 0.001 |
| 13 | Apigenin-*C*,*C*-dihexoside [f] | < LOQ | 0.12 ± 0.03 | 0.193 ± 0.003 | 0.3 ± 0.2 |
| 14 | Luteolin-*C*-hexoside-*C*-pentoside isomer [e] | 4.02 ± 0.07 | 5.1 ± 0.2 | 3.78 ± 0.06 | 3 ± 1 |
| 15 | Luteolin-*C*,*C*-dipentoside isomer [e] | 0.033 ± 0.009 | < LOQ | - | < LOD |
| 16 | Ferulyol quinic acid isomer [c] | - | 0.12 ± 0.03 | < LOD | < LOD |
| 17 | *p*-Coumaric acid | 0.104 ± 0.009 | 1.6 ± 0.6 | 2.1 ± 0.3 | 3 ± 1 |
| 18 | Apigenin-*C*-hexoside-*C*-pentoside isomer [f] | 4.76 ± 0.06 | 1.0 ± 0.2 | 7.0 ± 0.1 | 6.65 ± 0.07 |
| 19 | Luteolin-6-*C*-glucoside | 2.9 ± 0.1 | 6.0 ± 0.5 | 12.1 ± 0.9 | 3 ± 2 |
| 20 | Ferulic acid | - | 0.17 ± 0.05 | - | 0.7 ± 0.7 |
| 22 | Luteolin-*O*-desoxyhexosyl-*C*-hexoside [e] | 4.7 ± 0.2 | 5.9 ± 0.1 | 1.38 ± 0.07 | 1.0 ± 0.2 |
| 23 | Luteolin-*C*,*C*-dipentoside isomer [e] | 3.4 ± 0.1 | 0.39 ± 0.03 | 0.72 ± 0.03 | 0.65 ± 0.02 |
| 24 | Apigenin-8-*C*-glucoside [f] | 0.199 ± 0.005 | 0.12 ± 0.02 | 0.272 ± 0.006 | 0.13 ± 0.03 |
| 25 | *o*-Hydroxy benzoic acid | < LOQ | < LOQ | < LOQ | 0.072 ± 0.001 |
| 26 | Apigenin-*C*-hexoside isomer [f] | 0.359 ± 0.007 | 0.2 ± 0.1 | 2.27 ± 0.04 | 0.7 ± 0.2 |
| 27 | Luteolin-*O*-rutinoside [g] | 3.7 ± 0.1 | 1.3 ± 0.6 | 0.69 ± 0.04 | - |
| 28 | Luteolin-*O*-hexoside-*C*-pentoside isomer [e] | 1.00 ± 0.04 | 0.16 ± 0.04 | 0.27 ± 0.03 | - |
| 29 | Luteolin | < LOD | < LOQ | < LOD | < LOD |
| 30 | Tricin [h] | 0.62 ± 0.05 | 3.3 ± 0.7 | 4.47 ± 0.09 | 8 ± 2 |
| | Hydroxy benzoic acids | 21 ± 3 | 12.9 ± 0.9 | 5.2 ± 0.6 | 7.5 ± 0.9 |
| | Flavones | 26 ± 1 | 24 ± 2 | 33 ± 4 | 24 ± 3 |
| | Total compounds, mg compound/g extract | 47 ± 2 | 36 ± 2 | 38 ± 38 | 31 ± 2 |

[a] Amounts expressed as equivalents of 3-caffeoyl quinic acid (≥99%). [b] Amounts expressed as equivalents of *p*-hydroxy benzoic acid (≥99%). [c] Amounts expressed as equivalents of ferulic acid (≥99%). [d] Amounts expressed as equivalents of *p*-coumaric acid (≥98%). [e] Amounts expressed as equivalents of luteolin-6-*C*-glucoside (≥98%). [f] Amounts expressed as equivalents of apigenin-8-*C*-glucoside (95%). [g] Amounts expressed as equivalents of kaempferol-3-*O*-rutinoside (≥98%). [h] Amounts expressed as equivalents of quercetin (≥98%).
LOD: Limit of detection. LOQ: Limit of quantification.

**Table S13.** Results of the analysis of variance used to evaluate the effect of plant material, prior to distillation or biomass waste, on the chemical composition of hydroalcoholic extracts of Java type citronella and palmarosa.

| Compound | mg compound/g extract | | | | | | | |
|---|---|---|---|---|---|---|---|---|
| | Java type citronella | | | | Palmarosa | | | |
| | Before distillation | Residual biomass | F | *p* | Before distillation | Residual biomass | F | *p* |
| Ferulyol quinic acid isomer | B | A | 1426.12 | < 0.0001 | B | A | 1288.26 | < 0.0001 |
| Luteolin-*C*-hexoside-*C*-pentoside isomer | A | A | 1.88 | 0.2423 | A | A | 0.23 | 0.6541 |
| Apigenin-*C*-hexoside-*C*-pentoside isomer | B | A | 465.55 | < 0.0001 | A | A | 1.54 | 0.2819 |
| Luteolin-6-*C*-glucoside | A | B | 117.73 | 0.0017 | B | A | 33.83 | 0.0043 |
| Luteolin-*O*-desoxyhexosyl-*C*-hexoside | A | B | 41.54 | 0.0076 | B | A | 7.77 | 0.0494 |
| Tricin | A | B | 37.99 | 0.0035 | A | B | 8.69 | 0.0420 |

Different letters in the column indicate significant differences. A *p* value < 0.05 indicates that the effect is significant with a 95% confidence level. F-value.

**Table S14.** Detection and quantification limits, obtained by UHPLC-ESI$^{+/-}$-Orbitrap-MS, operated in SIM mode, of the standard compounds used in this study.

| Compound | Linear equation | $R^2$ | LOD, mg/kg | LOQ, mg/kg |
|---|---|---|---|---|
| *p*-Hidroxy benzoic acid | y = 4138252x+94396 | 0.9948 | 0.07 | 0.22 |
| *p*-Coumaric acid | y = 11327904x+178822 | 0.9969 | 0.05 | 0.16 |
| Caffeic acid | y = 36645495x+919453 | 0.9939 | 0.08 | 0.25 |
| Ferulic acid | y = 7760858x+93946 | 0.9986 | 0.03 | 0.12 |
| 3-Caffeoyl quinic acid | y = 12516386x+2256608 | 0.9983 | 0.04 | 0.13 |
| Luteolin-6-*C*-glucoside | y = 15143634x+95125<br>y = 19536796x-5177766 | 0.9974<br>0.9903 | 0.04 | 0.15 |
| Kaempferol-3-*O*-rutinoside | y = 9425233x+2732818 | 0.9916 | 0.02 | 0.09 |
| Luteolin | y = 70907433x+1810057 | 0.9972 | 0.05 | 0.17 |
| Apigenin-8-*C*-glucoside | y = 29902795x-234075<br>y = 24612203x+11476834 | 0.9964<br>0.9921 | 0.05 | 0.20 |
| Quercetin | y = 7706935x+ 573733 | 0.9913 | 0.08 | 0.25 |

**Table S15.** Results of the analysis of variance used to evaluate the effect of plant material, prior to distillation or biomass waste, on the antioxidant activities of hydroalcoholic extracts of Java type citronella and palmarosa.

| Compound | Antioxidant activities | | | | | | | |
|---|---|---|---|---|---|---|---|---|
| | Java type citronella | | | | Palmarosa | | | |
| | Before distillation | Residual biomass | F | *p* | Before distillation | Residual biomass | F | *p* |
| ORAC | A | A | 0.92 | 0.3742 | A | A | 3.36 | 0.1165 |
| $ABTS^{+\bullet}$ | A | A | 8.54 | 0.0614 | B | A | 223.62 | 0.0006 |

Different letters in the column indicate significant differences. A *p* value < 0.05 indicates that the effect is significant with a 95% confidence level. F-value.

**Table S16.** Variable loadings for the principal components of *C. martinii* EOs composition data.

| Essential oil constituent | Principal components (% variance explanation) | | | | |
|---|---|---|---|---|---|
| | **1 (91.296%)** | **2 (6.296%)** | **3 (1.864%)** | **4 (0.297%)** | **5 (0.248%)** |
| β-Myrcene | 0.000 | 0.029 | 0.227 | 0.157 | 0.108 |
| (*Z*)-β-Ocimene | -0.005 | 0.009 | 0.019 | -0.113 | 0.023 |
| (*E*)-β-Ocimene | -0.034 | -0.033 | 0.381 | -0.789 | 0.409 |
| Nerol | -0.005 | 0.009 | 0.124 | 0.098 | -0.306 |
| Neral | 0.000 | -0.005 | 0.055 | 0.113 | 0.056 |
| Linalool | -0.034 | -0.018 | 0.830 | 0.333 | 0.004 |
| Geraniol | -0.989 | 0.140 | -0.038 | 0.009 | -0.015 |
| Geranial | -0.007 | -0.006 | 0.018 | 0.434 | 0.612 |
| Geranyl acetate | -0.138 | -0.988 | -0.018 | 0.019 | -0.040 |
| (*E*)-β-Caryophyllene | 0.001 | -0.022 | -0.005 | 0.017 | 0.269 |
| Caryophyllene oxide | -0.002 | -0.005 | 0.076 | 0.036 | -0.101 |
| (2*E*,6*Z*-)-Farnesol | -0.008 | -0.029 | -0.296 | 0.128 | 0.514 |

**Table S17.** Variable loadings for the principal components of *C. winterianus* EOs composition data.

| Essential oil constituent | Principal components (% variance explanation) | | | | |
|---|---|---|---|---|---|
| | **1 (86.067%)** | **2 (9.413%)** | **3 (3.533%)** | **4 (0.633%)** | **5 (0.353%)** |
| Limonene | 0.027 | -0.084 | 0.076 | 0.147 | 0.329 |
| Linalool | 0.014 | -0.036 | 0.018 | 0.021 | 0.012 |
| Citronellal | 0.935 | 0.305 | -0.163 | 0.067 | -0.038 |
| Citronellol | 0.186 | -0.833 | -0.505 | -0.034 | -0.038 |
| Geraniol | 0.294 | -0.435 | 0.835 | -0.025 | 0.050 |
| Geranial | 0.000 | -0.002 | 0.020 | 0.027 | -0.078 |
| Citronellyl acetate | -0.027 | -0.061 | 0.035 | 0.164 | -0.688 |
| Eugenol | 0.016 | -0.014 | 0.046 | -0.001 | -0.077 |
| Geranyl acetate | -0.011 | -0.028 | 0.087 | 0.209 | -0.519 |
| β-Elemene | 0.006 | -0.004 | 0.010 | -0.100 | -0.043 |
| Germacrene D | 0.017 | -0.039 | -0.003 | -0.135 | -0.085 |
| δ-Cadinene | -0.005 | 0.028 | 0.002 | -0.085 | -0.128 |
| Elemol | 0.022 | 0.075 | 0.058 | -0.590 | -0.295 |
| Germacrene D-4-ol | 0.046 | -0.025 | -0.020 | -0.697 | 0.066 |
| α-Cadinol | -0.001 | 0.041 | 0.001 | -0.179 | -0.122 |

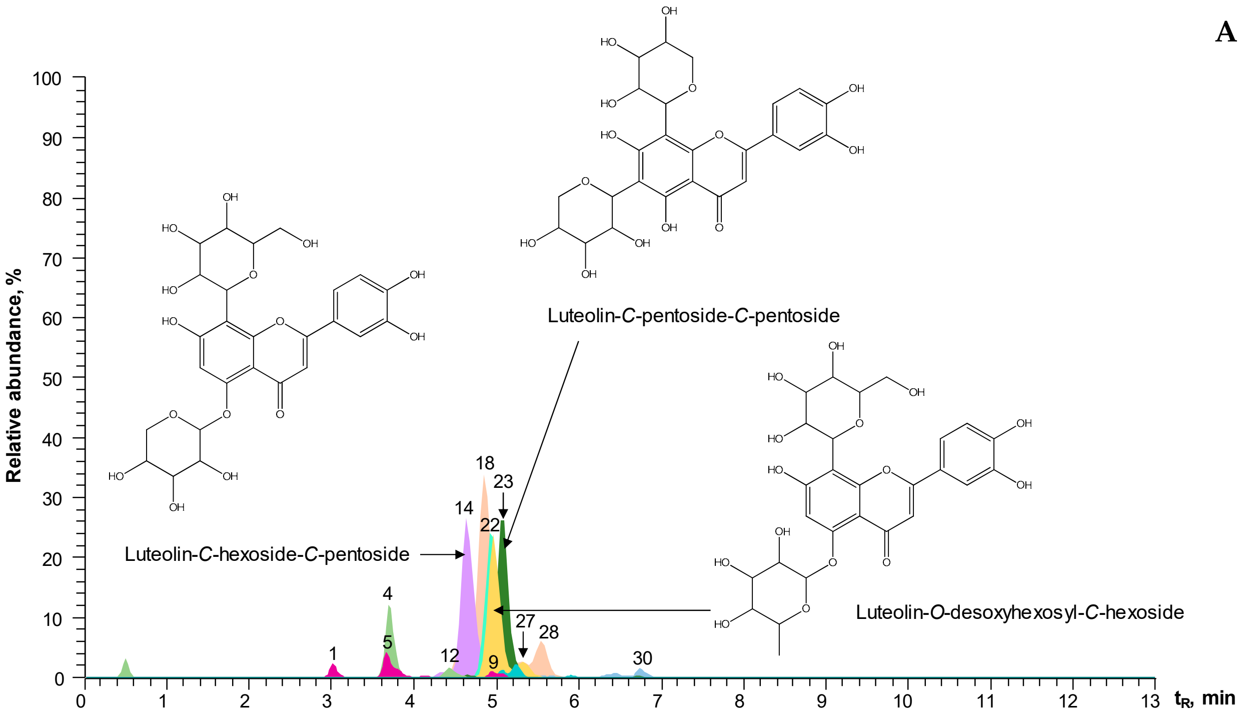


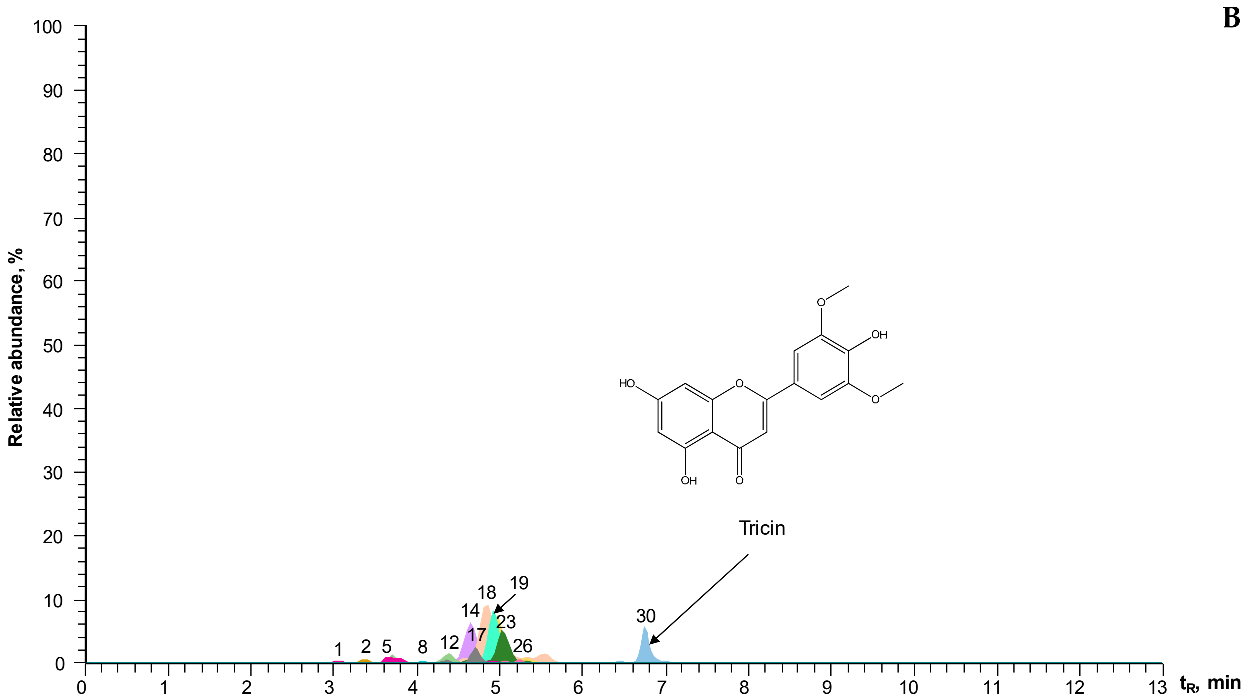


**Figure S1.** Extracted ion currents (EICs), obtained by UHPLC-ESI$^{+/-}$-Orbitrap-MS, operated in SIM mode, of the protonated $[M + H]^+$ and deprotonated $[M - H]^-$ molecules of the substances present in the *C. winterianus* solvent extracts obtained from: **A.** Prior to distillation plant material and **B.** Biomass waste. Scale 6.8 x $10^7$. See peak identification in **Table S6**.

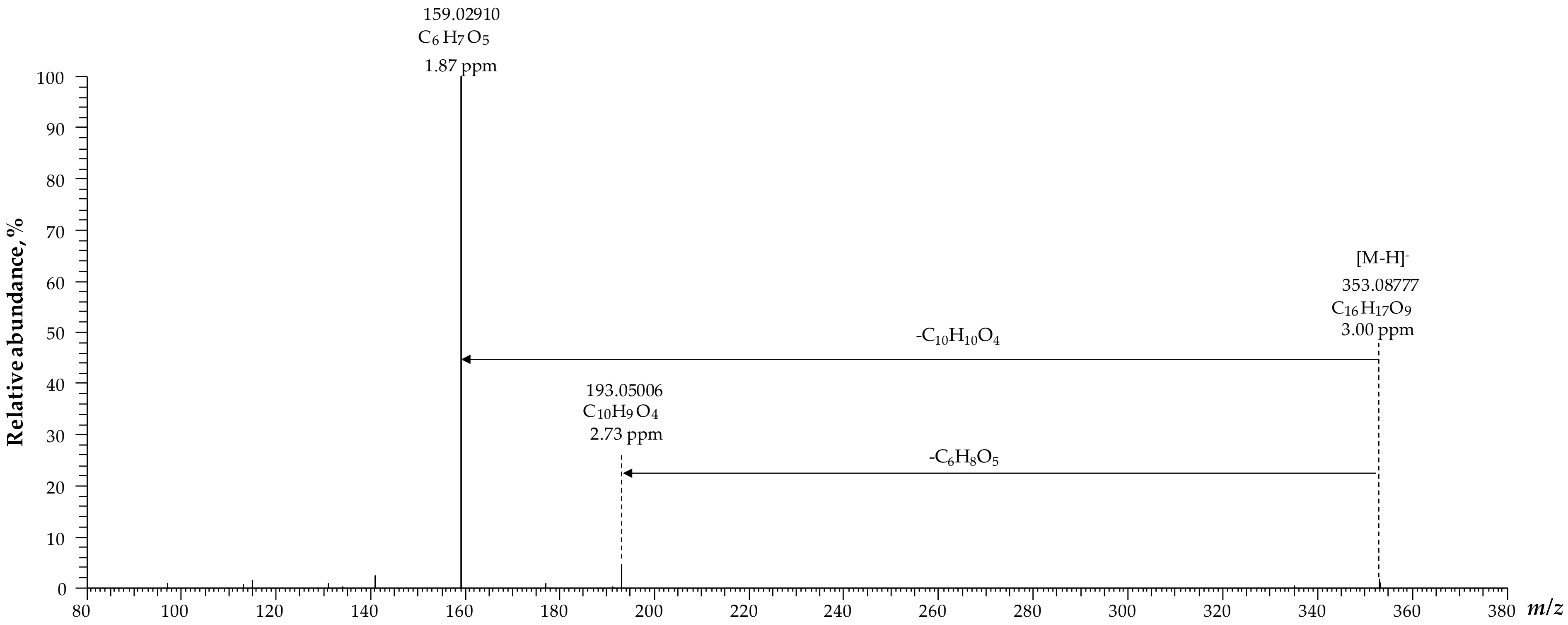


**Figure S2.** Mass spectrum, obtained by UHPLC-ESI+-Orbitrap-MS (HCD, 10 eV), of the deprotonated molecule $[M-H]^-$ of compound ($C_{16}H_{18}O_9$) present in Java type citronella and palmarosa solvent extracts, isolated from residual biomass. SIM-EIC of the ion at *m/z* 353.08777 $[M-H]^-$ and the formation of its main products. The Δ ppm value measures the approximation error in the measured mass compared to the expected or theoretical mass. To calculate the ppm, one must use the formula: Δ ppm = [(theoretical *m/z* value – experimental *m/z* value)/theoretical *m/z* value] × $10^6$.

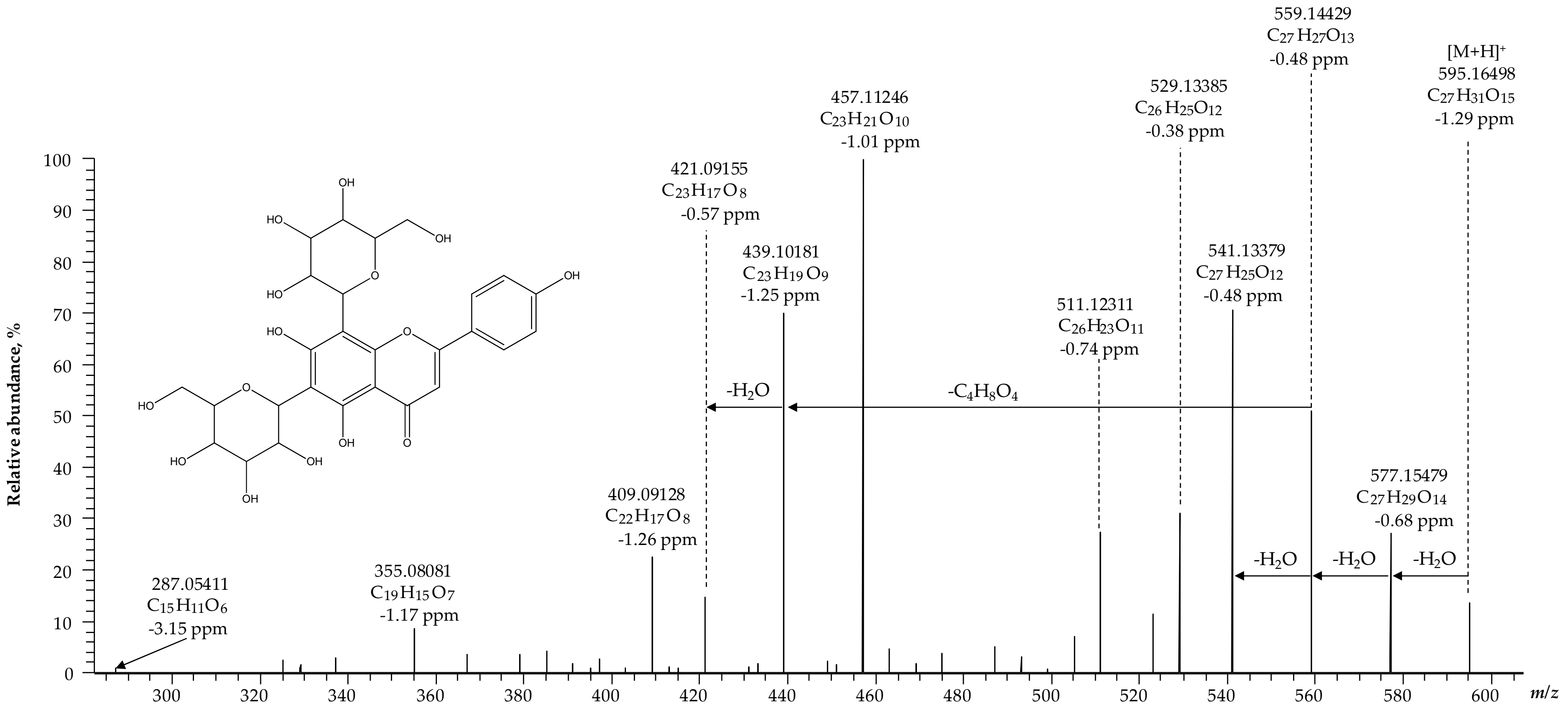


**Figure S3.** Mass spectrum, obtained by UHPLC-ESI+-Orbitrap-MS (HCD, 20 eV), of apigenin-*C,C*-dihexoside present in Java type citronella and palmarosa hydroalcoholic extracts, isolated from residual biomass. SIM-EIC of the ion at *m/z* 595.16498 $[M + H]^+$ and the formation of its main products. The Δ ppm value measures the approximation error in the measured mass compared to the expected or theoretical mass. To calculate the ppm, one must use the formula: Δ ppm = [(theoretical *m/z* value – experimental *m/z* value) ⁄ theoretical *m/z* value] × $10^6$.

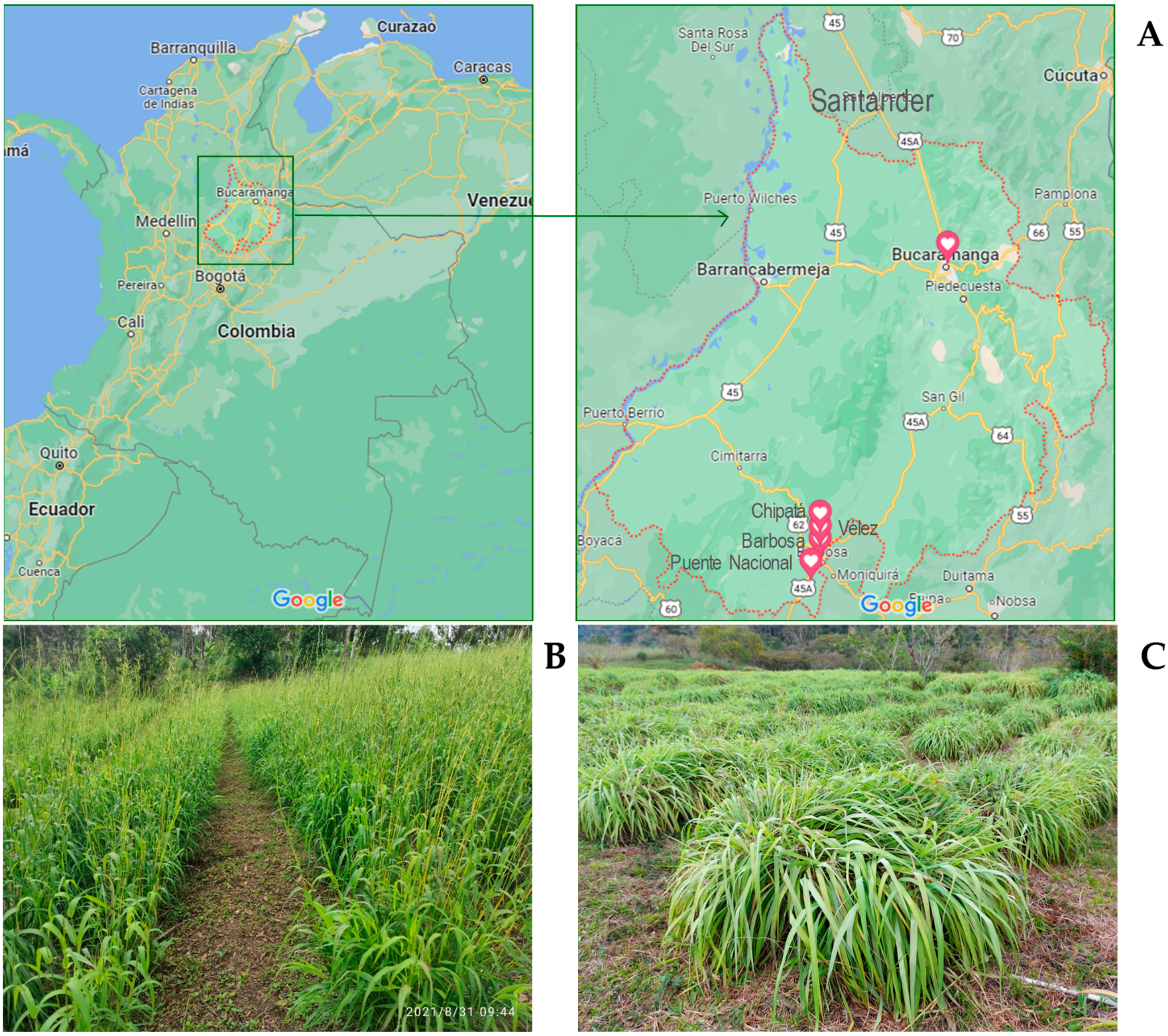


**Figure S4**. Geographical location of citronella and palmarosa crops. **A.** Adapted from Google-Maps. **B.** Palmarosa (*C. martinii*) plantation and **C.** Citronella (*C. winterianus*) plantation in the experimental plots at the municipality of Barbosa (Santander). 2021, August 31.
